# AI-driven advances in plant biotechnology: sharpening the edge of plant tissue culture and genome editing

**DOI:** 10.3389/fpls.2025.1718810

**Published:** 2025-12-10

**Authors:** Muralikrishna Narra, Anamika Ray, Brittany Polley, Hui Yang, Pankaj K. Bhowmik

**Affiliations:** 1 Aquatic and Crop Resource Development, National Research Council of Canada (NRC), Saskatoon, SK, Canada; 2 Anandi Botanicals Inc., Lethbridge, AB, Canada

**Keywords:** artificial intelligence, plant tissue culture, genome editing, machine learning, deep learning, CRISPR

## Abstract

The advent of artificial intelligence (AI) holds great promise for revolutionizing the fields of plant tissue culture and genome editing. Plant tissue culture is recognized as a powerful tool for rapid multiplication and crop improvement. However, the complex interactions between genetic and environmental factors generate large volumes of data, posing challenges for traditional statistical analysis methods. To address this, researchers are now employing machine learning (ML)-based and artificial neural networks (ANN) approaches to predict and optimize *in vitro* culture protocols thereby improving precision, sustainability, and efficiency. Integrating AI technologies such as machine learning (ML), artificial neural networks (ANN), and deep learning (DL) can significantly advance the development of data-driven models for CRISPR/Cas9 genome editing. Today, AI-driven methods are routinely applied to enhance precision in predicting on- and off-target sequence locations and editing outcomes. Additionally, predicting protein structures can provide a directed evolution framework that facilitates the creation of improved gene editing tools. However, the application of AI-based CRISPR modeling in plants is not yet fully explored. In this context, we aim to examine representative ML/DL/ANN models of CRISPR/Cas based editing employed in various organisms. This review significantly compiles a diverse set of studies and provides a clear overview of how AI is transforming the fields of plant tissue culture and genome editing. It emphasizes AI’s potential to increase the efficiency and precision of biotechnological practices, making them more accessible and cost-effective. While outlining current findings, the paper sets the stage for future research, encouraging further exploration into the integration of AI with plant biotechnology.

## 1 Introduction

In recent years, plant biotechnology has made significant strides, notably by the integration of artificial intelligence (AI) with plant tissue culture and genome editing, that represents a transformative advancement in plant biotechnology, offering powerful tools to accelerate crop improvement. Plant tissue culture and CRISPR/Cas genome editing have each revolutionized plant breeding and genetic engineering (Ibrahim et al., 2023). Incorporating AI techniques further enhances these technologies by enabling faster, more accurate, and cost-effective optimization of culture protocols and precise genetic modifications. AI models excel at deciphering complex, non-linear patterns in large biological datasets, enhancing predictions of growth responses, culture conditions, and editing outcomes (Kaushik et al., 2025). The continued advancement and adoption of AI methodologies are essential to overcoming existing technical and biological limitations. Harnessing the full potential of AI, in conjunction with plant tissue culture and genome editing, will drive the development of sustainable, precision agriculture solutions, supporting global food security, promoting environmental sustainability, and ushering in a new era of innovation in plant sciences.

Plant tissue culture is the sterile cultivation of plant explants on defined media under controlled conditions, leveraging totipotency, the ability of a single cell to regenerate into a whole plant (Phillips and Garda, 2019). It is fundamental to micropropagation, crop improvement, metabolite production, and conservation. To achieve efficient *in vitro* regeneration, factors such as explant type, nutrient composition and growth regulators must be carefully optimized (Loyola-Vargas and Ochoa-Alejo, 2018). Traditional statistical methods are limited in handling the complexity and non-linearity of *in vitro* systems, making optimization time-consuming and labor-intensive (Niazian and Niedbała, 2020). Artificial intelligence, especially machine learning and artificial neural networks, enables reliable modeling of complex biological interactions using experimental data. It facilitates accurate prediction and optimization of plant tissue culture stages, while reducing time, cost, and experimental load (Hesami et al., 2020a). The CRISPR/Cas system has emerged as a leading genome engineering platform due to its programmability and targeting flexibility (Liu et al., 2022a; Liu et al., 2022b). Its broad applicability has enabled its use across a wide range of applications, from gene disruption and modification to gene regulation and RNA editing. Despite challenges like off-target effects and variable efficiency, the focus has shifted from feasibility to optimization (Ran et al., 2013, 2015). Machine learning (ML)/Deep learning (DL) offers powerful methods for modeling complex biological systems. By learning patterns from data, AI enables accurate prediction of CRISPR editing outcomes, surpassing the limits of traditional experimentation (Chuai et al., 2017). The integration of deep learning into genomics and genetic engineering presents new opportunities for precision and innovation in gene editing (Eraslan et al., 2019).

While previous reviews have acknowledged the promise of AI)in plant biotechnology, most focus narrowly either on ML applications in plant tissue culture or on CRISPR/Cas-based genome editing in non-plant systems. For instance, certain studies (Hesami and Jones, 2021; Ibrahim et al., 2023; Kaushik et al., 2025) offer valuable overviews of AI in tissue culture optimization but do not address the convergence of AI with gene editing platforms like CRISPR/Cas9. This review is uniquely positioned at the intersection of two critical domains and emphasizes the integrative role of AI across plant tissue culture and genome editing. In addition to summarizing current models and algorithms, this review outlines challenges in data availability, model interpretability, and experimental validation, and proposes concrete future research directions to bridge these gaps. This positions the review as both a resource and a roadmap for researchers aiming to operationalize AI in plant biotechnology.

## 2 Machine learning-based approaches in plant tissue culture

Plant tissue culture (micropropagation or *in vitro* cell and tissue culture), is a technique used to grow plants in a nutrient-rich medium under controlled, sterile conditions. It begins with culturing various explants such as leaves, stems, or roots, which due to their totipotency capacity, can regenerate into whole plants and produce multiple plantlets (Loberant and Altman, 2010). This technique has diverse applications, including mass propagation of elite plants, genetic modification, germplasm conservation, and production of disease-free plants (Brown and Thorpe, 1995). *In vitro* cultures are influenced by several factors, including nutrient media composition, plant genotype, explant type and age, plant growth regulators (PGRs), and phytohormone concentrations (Sudheer et al., 2022). Traditional regression methods and analysis of variance (ANOVA) have been employed for analyzing the data. However, the complexity and non-linearity of biological systems often limit the effectiveness of these techniques (Compton, 2024). In contrast, AI and computer-assisted tools can process continuous, binomial, discrete, and incomplete data, even those generated through unstructured, trial-and-error experiments. This ultimately reduces the need for extensive laboratory experimentation, saving time and resources in optimizing conditions for *in vitro* culture (Sarker, 2022).

**Figure 1:**
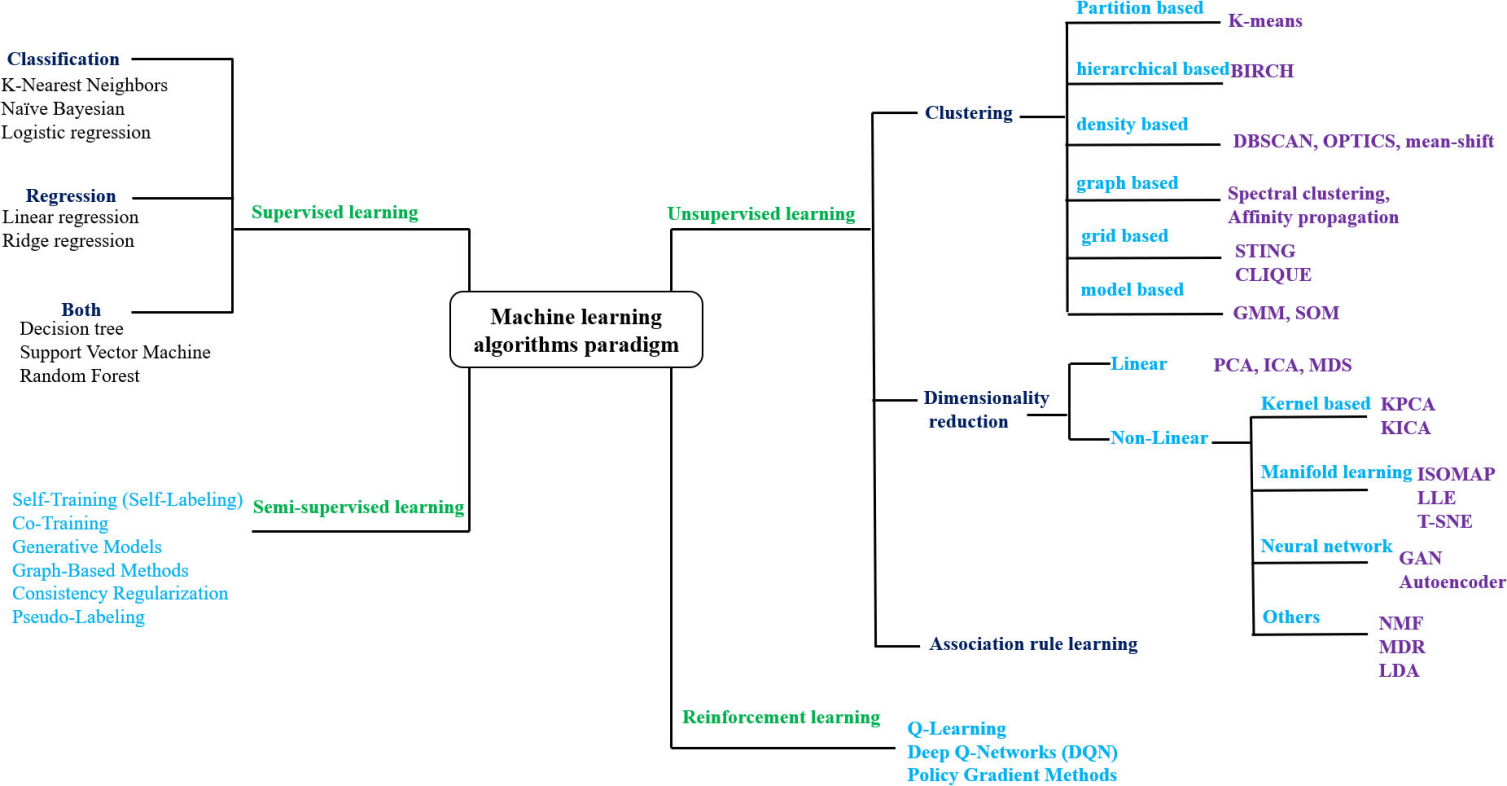
Taxonomy of machine learning algorithm paradigms. BIRCH, Balanced Iterative Reducing and Clustering Using Hierarchies; DBSCAN, Density-Based Spatial Clustering of Applications with Noise; OPTICS, Ordering Points To Identify the Clustering Structure; STING, STatistical INformation Grid; CLIQUE), CLustering In QUEst; GMM, Gaussian Mixture Models; SOM, Self-Organizing Map; PCA, Principal Component Analysis; ICA, Independent Component Analysis; MDS, MultiDimensional Scaling; KPCA, Kernel PCA; KICA, Kernel ICA; ISOMAP, ISOmetric feature MAPping; LLE, Locally Linear Embedding; t-SNE, t-distributed Stochastic Neighbor Embedding; NMF, N on-negative Matrix Factorization; MDR, Multifactor Dimensionality Reduction; LDA, Latent Dirichlet Allocation; GAN, Generative Adversarial Network.

ML has emerged as a powerful tool for addressing this challenge, enabling computers to learn from data and make accurate predictions and classifications (Ray, 2019). ML techniques include supervised learning, where models are trained on labeled datasets for precise prediction and classification (Alloghani et al., 2020); unsupervised learning, which identifies patterns in unlabeled data for clustering and analysis (Alloghani et al., 2020); reinforcement learning, which improves performance through trial-and-error interactions with the environment (Szepesvári, 2010); and semi-supervised learning, that integrates limited labeled data with abundant unlabeled data to enhance model accuracy, particularly when labeling is costly or time-consuming (Zhu and Goldberg, 2009) (**Figure 1**). In plant tissue culture research, recent advances have leveraged ML to interpret complex, nonlinear data. Hybrid approaches combining ML with optimization algorithms have been used to analyze the relationships between variables. As a result, data-driven approaches are increasingly used to model and optimize tissue culture conditions, including media composition and other critical parameters (Sarker, 2022). A comprehensive literature review was conducted to collect the data specific to plant tissue culture. Using online databases, google scholar, pubmed and scopus were searched for relevant studies with keywords such as “*MACHINE LEARNING IN PLANT TISSUE CULTURE*”, “*ANN IN PLANT TISSUE CULTURE*”. Selected peer-reviewed publications with machine learning application in plant tissue culture techniques were screened and considered for writing this review article (**Table 1**).

**Table 1:** Applications of machine learning-based approaches/models used in plant tissue culture and other prediction studies.

Machine learning Types		Algorithm/Model	Plant species	Application/Prediction output	Performance metric values	Reference
Supervised Learning	**Regression**	Linear Regression	*Pinus taeda*	Organogenesis, Nutrient management	R^2^: 0.8 MRA, 0.83 ANN; RMSE: 13.32 MRA, 12.34 ANN	Barone, 2019
			*Punica granatum*	Effective in fine-tuning plant growth regulator concentrations	R^2^: 0.319 ENMLR, 0.358 XGB; RMSE: 0.773 ENMLR, 0.637 XGB	Zarbakhsh et al., 2024
			*Juglans regia*	Enhancing shoot proliferation	R^2^: 0.428 MLR, 0.695 MLPNN; RMSE: 3.313 MLR, 1.658 MLPNN	Sadat-Hosseini et al., 2022
			*Lilaeopsis brasiliensis*	Identifying influential variables	R^2^: 0.111 MLR, 0.206 MLP; RMSE: 3.771 MLR, 3.565 MLP	Ali and Aasim, 2024
	**Classification**	K-Nearest Neighbors (KNN)	*Juglans regia*	*In vitro* proliferation	R^2^: 0.428 MLR, 0.672 KNN; RMSE: 3.313 MLR, 1.756 KNN	Sadat-Hosseini et al., 2022
			*Miscanthus sinensis*	Seed germination	ROC KNN: 0.89	Awty-Carroll et al., 2018
			*Cucumis sativus*	Classified environmental stress levels	Accuracy: 0.93 KNN; F1 score: 0.93	Lee et al., 2025
		Naive Bayesian	*Musa textilis*	Tissue culture contamination	Accuracy: 92%; AUC: 0.85–0.92	Malangsa and Maravillas, 2017
			*Helianthus annuus*	Species classification in plant genetic resources	Accuracy: 0.96 3-NN	van Bemmelen van der Plaat et al., 2021
			*Arabidopsis thaliana, Glycine max, Oryza sativa & Prunus persica*	To identify plant microRNAs	Prediction: *Arabidopsis thaliana* (43%), *Glycine max* (24%), *Oryza sativa* (30%), *Prunus persica* (18%)	Douglass et al., 2016
		Logistic Regression	*Pistacia vera*	Identifying optimal conditions for regeneration	Accuracy: 85–90%	Onay et al., 2000
			*Pinaceae* spp.	Classifying plant embryos	Precision: 0.75–0.88	Jones, 2012
	**Regression and Classification**	Support Vector Machines (SVM)	*Chrysanthemum morifolium*	Somatic embryogenesis	R^2^: 0.99 SVM, 0.91 MLP; RMSE: 0.94 SVM, 2.07 MLP	Hesami et al., 2020a
			*Cannabis sativa*	Optimization of callus growth	R^2^: 0.759 SVM, 0.718 RF; RMSE: 0.121 SVM, 0.098 RF	Hesami and Jones, 2021
			*Lycium* spp.	Impact of cadmium stress on micropropagation	R^2^: 0.93 SVM, 0.94 MLP, 0.95 RF; RMSE: 0.08 SVM, 0.07 MLP, 0.07 RF	Isak et al., 2024
		Decision trees	*Sorghum bicolor*	Direct organogenesis	R^2^: 0.799 MLP, 0.779 RF, 0.768 XGB	Aasim et al., 2023
			*Prunus armeniaca*	Refined media composition & other parameters affecting shoot growth	Pearson correlation coefficient: 0.70 (p<0.01)	Kovalchuk et al., 2017
			*Corylus avellana*	Identified mineral concentrations affecting shoot growth parameters	Pearson correlation coefficient: 0.661 (p<0.01)	Akin et al., 2017
		Random Forest (RF)	*Cannabis sativa*	*In vitro* germination	Accuracy: 0.88 MLP, 0.79 SVC, 0.73 XGB, 1.00 RF; F1 score: 0.84 MLP, 0.77 SVC, 0.79 XGB, 1.00 RF	Aasim et al., 2022
			*Hemianthus callitrichoides*	Formulation of media composition	R^2^: 0.885 MLP, 0.890 RF, 0.859 XGB; RMSE: 0.285 MLP, 0.271 RF, 0.348 XGB	Özcan et al., 2023
			*Passiflora caerulea*	Shoot regeneration, identifying precise hormonal combination	R^2^: 0.99 GRNN, 0.96 RF; RMSE: 3.08 GRNN, 3.12 RF	Jafari and Daneshvar, 2023
			*Punica granatum*	Shoot proliferation predictions	R^2^: 0.319 ENMLR, 0.358 XGB; RMSE: 0.773 ENMLR, 0.637 XGB	Zarbakhsh et al., 2024
			*Cannabis sativa*	Callogenesis	R^2^: 0.759 SVM, 0.718 RF; RMSE: 0.121 SVM, 0.098 RF	Hesami and Jones, 2021
			*Fragaria × ananassa*	Genotype-specific modeling	R^2^: 0.55 MLP, 0.59 SVM, 0.78 RF; RMSE: 0.91 MLP, 0.76 SVM, 0.57 RF	Şimşek, 2024
*Unsuper‐vised Learning		K-means (KAT4IA)	*Zea mays*	Field-based phenotyping	k-means cluster analysis	Guo et al., 2021
		K-means clustering and affinity propagation	*Medicago truncatula*	Identifying stress markers	Gaussian Process 2-Sample Test, k-means cluster analysis	Dickinson et al., 2018
*Reinforcement Learning		Mechanics and Leaves (MeLe)	*Condylocarpon guianense*	Adaptive complexity of plant morphology	total length shoot sample: 0.868 meters	Nasti et al., 2024
*Semi‐supervised Learning		DM_CorrMatch	*Brassica napus*	Rapeseed inflorescence segmentation	Intersection over Union (IoU): 0.886; Precision: 0.942; Recall: 0.940	Li et al., 2025
		PlantVillage dataset	Various spp.	Plant disease detection	single semi-supervised: 2.8%; iterative semi-supervised: 4.6%	Li and Chao, 2021
		U-Net	*Triticum* spp.	Head segmentation	Dice score: 0.89	Najafian et al., 2023

*Yet be expanded in the field of plant tissue culture.

R2, Coefficient of determination; RMSE, Root mean square error; MRA, Multiple regression analysis; ENMLR, Elastic net multivariate linear regression; XGB, Extreme gradient boosting; MLPNN, Multi-layer perceptron neural network; ROC, Receiver operating characteristic curves; AUC, Area under curve; 3-NN, 3-Nearest Neighbors; GRNN, generalized regression neural network, SVC, Support vector classification.

### 2.1 Supervised learning

**Figure 2:**
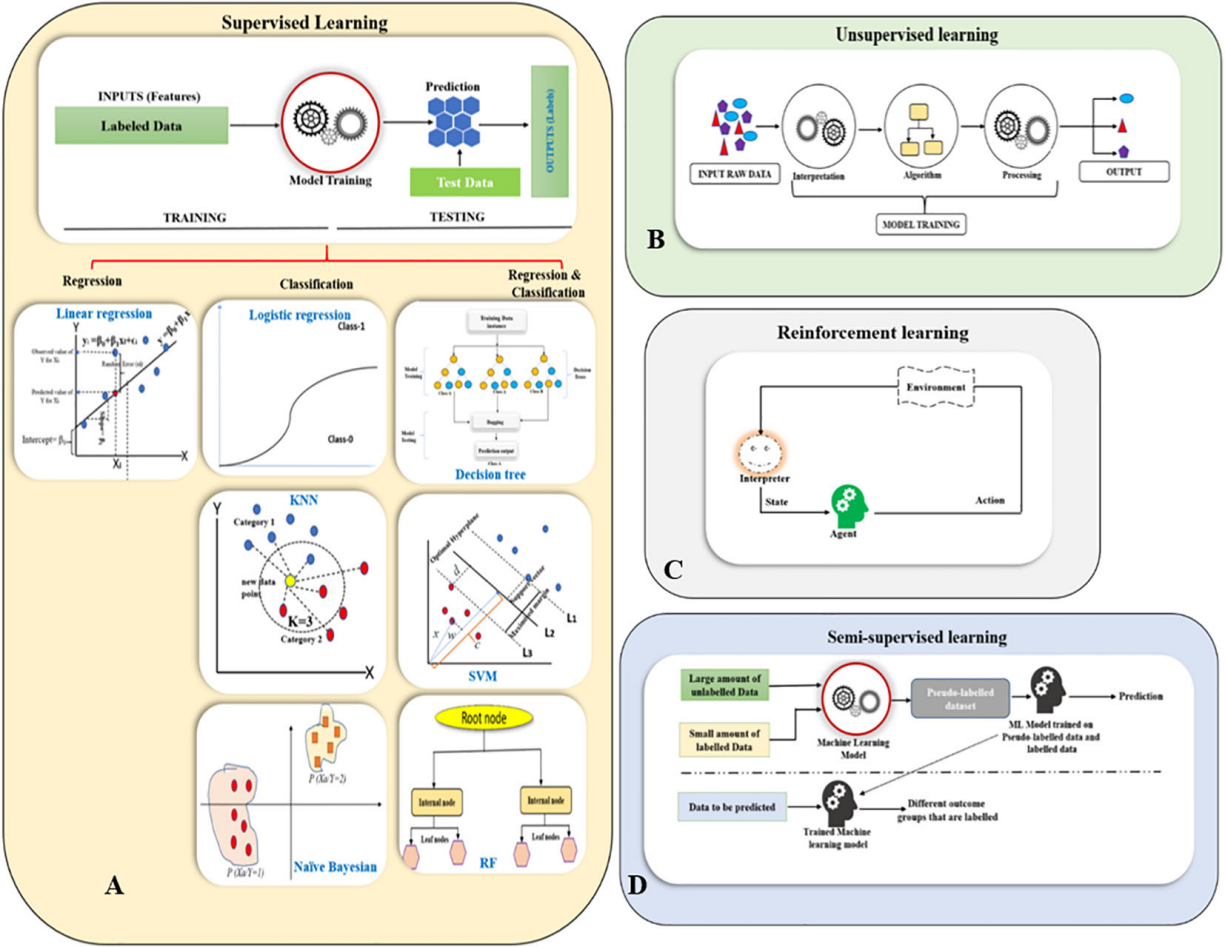
Overview of machine learning paradigms. **(A)** Supervised Learning (Linear Regression, Logistic Regression, Decision Trees, K-Nearest Neighbors (KNN), Support Vector Machines (SVM), Naïve Bayes, and Random Forests (RF); **(B)** Unsupervised Learning; **(C)** Reinforcement Learning; **(D)** Semi-supervised learning.

Supervised learning is a subset of machine learning that employs labeled datasets to train algorithm models for analysis and prediction in plant tissue culture research. The success of tissue culture is influenced by variety of factors, including sterilization techniques, plant genotype, growth conditions and media composition. Supervised learning algorithms help to model and predict these outcomes. The process begins with model training by using a dataset that includes input data (features) and corresponding output data (labels or target variables). Once the training is complete, the model is evaluated on a test dataset to assess its accuracy and performance. During the learning process, the algorithm analyzes the relationship between the input features and the output labels (Hastie et al., 2009a). The model’s performance is then fine-tuned by adjusting parameters and cross-validation to balance bias and variance, this sequentially helps to ensure that the model generalizes effectively to new, unseen data. Based on the nature of the output variables, supervised learning can be broadly categorized into (i) Regression, that deals with continuous, numerical outputs without predefined labels, for example, predicting growth rate, biomass accumulation, or shoot multiplication based on factors like temperature, light, or media compositions. (ii) Classification on the other hand, involves categorical outputs with defined labels, such as classifying tissue culture outcomes into categories like successful regeneration, no regeneration, or diseased (Hastie et al., 2009a) (**Figure 2A**).

#### 2.1.1 Linear regression

This is used to predict continuous variables based on one or more independent variables. It computes the linear relationship between the dependent variable and one or more independent features by fitting a linear equation to the observed data. The primary objective of linear regression is to find the best-fit line, which minimizes the error between predicted and actual values. Here the dependent variables (Y), the output you want to predict (e.g., plant growth rate, biomass) and independent variables (X), the factors affecting the output (e.g., temperature, humidity, light intensity) (Equation 1).

(1) $$y_{i}=\beta_{0}+\beta_{1}x_{i}+\epsilon_{i}$$

Where:

β_0_ is the intercept (value of y when x=0).

β_1_​ is the coefficient (effect of x on y).

ϵ**_i_** is the error term (difference between predicted and actual values).

ϵ**_i_** = y(predicted) – y**_i_**

where y(predicted) = β_0_+β_1_x**_i_**

The equation of this best-fit line represents the relationship between the dependent and independent variables, with the slope indicating how much the dependent variable changes for a unit change in the independent variable(s). Linear regression can be classified further into (i) simple linear regression, that models the relationship between one independent variable and one dependent variable by fitting a straight line. The line is defined by an intercept (β_0_) and a slope (β_1_), where the slope indicates the change in the dependent variable for each unit increase in the independent variable, and the intercept represents the expected value of the dependent variable when the independent variable is zero. The model aims to minimize the difference between actual and predicted values using a cost function, typically mean squared error (MSE) (Equation 2).

(2) $$MSE=\;1/N\sum_{i=1}^{n}{\left(y_{i}–\;(\beta_{0}+\beta_{1}x_{i})\right)}^{2}$$

Model performance is also evaluated using metrics like the R-squared (which shows the proportion of explained variance), residual standard error (RSE) and root mean squared error (RMSE). Valid regression requires assumptions such as linearity, independence, normality, and constant variance of residuals. (ii) multiple linear regression, extends this concept to include multiple independent variables (Equation 3):

(3) $$y_{i}=\beta_{0}+\beta_{1}x_{i\;}+\beta_{2}x_{ii\;}+\ldots .+\beta_{n}x_{n}\;+\;\epsilon_{i}$$

It follows the same assumptions as simple linear regression, with added concerns like multicollinearity, overfitting, and the need for feature selection. The bias-variance tradeoff is crucial where underfitting occurs when the model is too simple, while overfitting happens when the model captures noise instead of the true pattern, especially in high-dimensional or collinear datasets. Techniques like cross-validation, regularization, and careful feature engineering help strike the right balance. In summary, linear regression is a foundational tool in data science and machine learning, valued for its interpretability and effectiveness. It not only helps make accurate predictions but also lays the groundwork for more complex modeling approaches (Hastie et al., 2009b).

**Research application**

The reviewed studies collectively highlight the exponential use of integrated regression-based machine learning techniques in optimizing plant tissue culture protocols. In pomegranate, a combination of Bayesian-tuned ensemble stacking regression and non-dominated sorting genetic algorithm-II (NSGA-II) proved effective in fine-tuning plant growth regulators (PGR) concentrations, enhancing shoot proliferation while minimizing somaclonal variation (Zarbakhsh et al., 2024). Similarly, in *Pinus taeda*, multiple regression and neural networks revealed nitrogen concentration as a key factor in organogenesis, emphasizing the need for precise nutrient management (Barone, 2019). The comparative modeling in persian walnut (*Juglans regia*) revealed that although linear regression offers baseline predictions, advanced models like genetic programming (GEP) and multilayer perceptron neural network (MLPNN) deliver superior accuracy in forecasting *in vitro* proliferation. The optimization of macronutrient compositions for pear rootstocks leveraged both stepwise regression and AI methods to pinpoint critical factors influencing explant growth, showcasing the synergy between statistical and algorithmic tools (Sadat-Hosseini et al., 2022). Lastly, the application of response surface methodology and regression analysis in brazilian micro sword (*Lilaeopsis brasiliensis*) regeneration highlighted the practical utility of these models in identifying influential variables. Overall, these studies validate the robustness of regression and hybrid modeling approaches in refining tissue culture conditions across diverse plant species (Ali and Aasim, 2024).

#### 2.1.2 K-nearest neighbors

K-Nearest neighbors (KNN) machine learning algorithm was generally used for classification but can also be applied to regression tasks. It works by finding the “*K*” closest data points (neighbors) to a given input and makes predictions based on the majority class for classification or the average value for regression (Mucherino et al., 2009). Since KNN makes no assumptions about the underlying data distribution, it is considered as a non-parametric and instance-based learning method. K-nearest neighbors is also called a lazy learner algorithm because it does not learn from the training set immediately; instead, it stores the dataset and performs computations on it only at the time of classification. Cross-validation is a reliable method for choosing the optimal “*K*” in KNN by dividing the dataset into k parts, training on some, and testing on others, then selecting the k with the highest average accuracy. The k-nearest neighbors (KNN) algorithm functions with selecting an optimal value for *K*, which denotes the number of neighbors to consider when making predictions. Next, the algorithm calculates the distance between the target data point and all points in the training set, commonly using Euclidean distance as the measure of similarity, which is a straight-line distance between two points in a plane or space. It then identifies the “*K*” data points with the smallest distances to the target, designating these as the nearest neighbors (Equation 4) (James et al., 2021).

(4) $$Distance\;\left(x,X_{i}\right)=\sqrt{\sum_{j=1}^{d}{\left(xj-xij\right)}^{2}}$$

**Research application**

Optimizing plant tissue culture media is a complex process influenced by multiple factors including genotype, mineral nutrients, plant growth regulators (PGRs), and vitamins, often resulting in inefficiencies and physiological disorders like browning of callus, shoot tip necrosis and vitrification. In this context, predictive modeling using ML offers a promising solution. In walnut (*Juglans regia* L.) proliferation, three ML models, multi-layer perceptron neural network (MLPNN), KNN, and gene expression programming (GEP) were evaluated against multiple linear regression (MLR) for their predictive accuracy. All ML models outperformed MLR, with GEP showing the highest R² (0.802 in Chandler and 0.428 in Rayen varieties) and subsequently optimized using particle swarm optimization (PSO). This highlights GEP-PSO as a powerful hybrid tool, while MLPNN and KNN also demonstrated strong estimation abilities (Sadat-Hosseini et al., 2022). Similarly, in miscanthus seed germination, KNN improved phenotype scoring accuracy when compared to human assessments, especially under challenging conditions such as low germination rates and presence of mold. The model achieved a ROC-AUC of 0.69–0.7, improving to 0.89 on optimized image sets, confirming its utility for consistent, automated germination analysis (Awty-Carroll et al., 2018). In cucumber seedlings, ML models combined with image-based feature extraction (color, texture, morphology) effectively classified environmental stress levels. Among the tested models, KNN achieved the highest accuracy (94%), emphasizing its effectiveness in early stress detection and its potential application in precision agriculture for real-time crop monitoring (Lee et al., 2025).

#### 2.1.3 Naive Bayesian

Naive Bayesian is a classification algorithm that uses probability to predict which category a data point belongs to, assuming that all features are unrelated. The naive Bayesian classifier is based on bayes theorem, which describes the probability of an event, based on prior knowledge of conditions that might be related to the event (Webb, 2011) (Equation 5).

The general formula for Bayes’ theorem is:

(5) $$P\left(y/x\right)=\frac{P\left(X/y\right).P\left(y\right)\;}{P\left(X\right)\;}$$

Where:

P(y/x) is the posterior probability of class y given the features X.

P(x/y) is the likelihood, i.e., the probability of observing the features X given class y.

P(y)is the prior probability of class y.

P(x) is the evidence or the total probability of observing the features X under all classes.

The term “naive” comes from the assumption that all features are independent of each other, given the class label. This assumption simplifies the computation of the likelihood P(x/y), as we can decompose it into the product of individual feature probabilities. Naïve Bayesian classifiers can be broadly categorized based on the type of data they handle. (i) The gaussian naive Bayesian is used when the features are continuous and assumes that these features follow a gaussian (normal) distribution. In this case, the probability *P(y∣ X*) is estimated using the probability density function (PDF) of the normal distribution. (ii) The multinomial naïve Bayesian, is commonly used for text classification where the features are discrete counts, such as word frequencies in documents. It models the probability of each feature’s occurrence given a particular class using a multinomial distribution. (iii) Lastly, bernoulli naïve Bayesian is employed when the features are binary, representing the presence or absence of a feature (e.g., 0 or 1). It assumes that each feature follows a bernoulli distribution, where the outcomes are binary. Preprocessing steps like color normalization and data augmentation enhance naïve bayes performance by reducing variability and improving generalizability in image-based tissue culture analysis.

**Research application**

The performances of naïve bayes and KNN classifiers were compared for grading contamination in abaca tissue culture specimens. The methodology involved capturing images with a masking technique, extracting features from mean RGB values and binary images. Specimens were classified as healthy or contaminated, and classifier performance was evaluated using accuracy, precision, and recall. Naïve Bayes outperformed KNN, achieving 76% accuracy, compared to 68% for KNN at *K* = 3 and 58% at *K* = 7 (Malangsa and Maravillas, 2017). Different machine learning classifiers like random forest (RF), neighbor-joining (NJ), KNN, and naïve bayes (NB) were evaluated for species classification in plant genetic resources collections in sunflower. The authors found the KNN classifier to be the most reliable, especially for datasets with variability and uncertainty. The study also highlighted the importance of marker selection for improving classifier accuracy and introduced a method to enhance suboptimal datasets, particularly valuable for genebanks with limited high-quality references (van Bemmelen van der Plaat et al., 2021). Douglass et al. (2016) developed a naïve bayes classifier to identify plant microRNAs (miRNAs), which are key regulatory molecules in eukaryotes. Traditional methods, relying on stem-loop structures, often miss low-count miRNAs. Their probabilistic approach uses sequence length, observation counts, miRNA sequence presence, and other features, and was tested on small RNA data from soybean, peach, *Arabidopsis*, and rice.

#### 2.1.4 Logistic regression

Logistic regression is primarily used for classification tasks (Sperandei, 2014). Unlike linear regression, which predicts continuous values, logistic regression estimates the probability that an input belongs to a specific class. It is mainly employed for binary classification problems, where the output consists of two possible outcomes, such as Yes/No; True/False; or 0/1. The algorithm utilizes a sigmoid function to convert inputs into a probability between 0 and 1. In contrast, multinomial logistic regression is applied when the dependent variable has three or more unordered categories. It extends the binary logistic regression approach to accommodate multiple classes. Lastly, ordinal logistic regression is used when the dependent variable consists of three or more categories with a natural order or ranking, like ratings of “low,” “medium,” and “high.” This model accounts for the inherent order in the categories when making predictions. The logistic regression model is represented by the following equation (Equation 6):

(6) $$p\;\left(y=1/X\right)=\sigma \left(w^{T}X+b\right)$$

Where:

p(y=1/X) is the probability that the instance belongs to class 1 given the feature vector X.

w^T^X is the linear combination of the features (i.e., weights and features).

b is the bias term (also called the intercept).

Σ(sigma) is the sigmoid function that maps the linear combination to a probability.

To minimize the loss function, gradient descent is commonly used, iteratively adjusting the model parameters (weights and bias) to reduce the error. In addition to the primary cost function, regularization techniques like L1 (lasso) and L2 (ridge) regularization are often applied to prevent overfitting, especially in cases with high-dimensional data.

**Research application**

Linear logistic models were used to assess the significance of the treatments and identify optimal conditions for both processes, highlighting BAP’s superior role in regeneration. This study has explored the effects of 6-benzylaminopurine (BAP), abscisic acid (ABA), and sucrose on the germination and plantlet regeneration of pistachio somatic embryos. Germination rates increased with longer culture durations, with BAP and ABA concentrations influencing outcomes, while sucrose had little effect. Similarly, plantlet regeneration improved over time but was inhibited at higher BAP or ABA levels, with ABA reducing the likelihood of regeneration, particularly during extended maturation periods (Onay et al., 2000). A method for classifying plant embryos (Pseudotsuga and Pinus) based on quality using a penalized logistic regression (PLR) model is disclosed. First, image or spectral data are collected from plant embryos with known quality. Next, each data set is assigned a class label corresponding to the embryo’s quality. Then, metrics are calculated from these image or spectral data sets (Jones, 2012).

#### 2.1.5 Support vector machines

A support vector machine is a powerful ML-algorithm used for both classification and regression tasks. It works by finding the optimal line (or hyperplane) that separates data into distinct groups, while maximizing the distance between the closest data points (support vectors) of each group (Vapnik, 2000). A larger margin typically results in better generalization, allowing the model to perform well on new, unseen data. The optimal hyperplane, also known as the “hard margin,” is the one that maximizes this distance, ensuring a clear separation between the classes. A soft margin, on the other hand, allows for some misclassifications or violations of the margin, which helps improve generalization. This optimizes the following equation to balance margin maximization and penalty minimization (Equation 7).

(7) $$Objective\;Function\;=\;\left(\frac{1}{Margin}\right)+\lambda \sum penalty$$

The penalty for violations is typically the hinge loss, which behaves as follows, if a data point is correctly classified and lies within the margin, there is no penalty (loss = 0) and if a data point is misclassified or violates the margin, the hinge loss increases in proportion to the distance of the violation.

During the training phase, the algorithm is fed with labeled data points from both classes. This is for to determine the optimal hyperplane that maximizes the margin between the two classes, ensuring that no data points fall between the hyperplane and the support vectors. Once the model is trained, it enters the testing phase, where it is presented with new, unseen data points. The model then assesses the position of these points relative to the hyperplane and classifies them based on which side of the hyperplane they are located. A kernel is a function that allows support vector machines to handle non-linear data by implicitly mapping inputs to a higher-dimensional space. Common types include the linear kernel, suitable for linearly separable data; the polynomial kernel, which captures more complex relationships using polynomial functions; and the RBF (Radial basis function) kernel, which measures similarity based on the distance between data points.

**Research application**

Recent studies have demonstrated the effective application of SVM in plant tissue culture, particularly for modeling and optimizing processes such as somatic embryogenesis and callus development. Support vector regression (SVR) was employed to model somatic embryogenesis in chrysanthemum. The SVR model outperformed multilayer perceptron (MLP) models, achieving an R² value greater than 0.92. Furthermore, integrating SVR with the NSGA-II led to optimization of the culture medium, resulting in a 99.09% embryogenesis rate and an average of 56.24 somatic embryos per explant (Hesami et al., 2020a). SVM in combination with random forest (RF) and genetic algorithm (GA) to model and optimize callus growth in *Cannabis sativa*. This hybrid approach improved prediction accuracy and provided valuable insights into the influence of various factors on callus development (Hesami and Jones, 2021). More recently, a study by Isak et al. (2024) utilized multiple machine learning algorithms, including SVM, to evaluate the impact of cadmium stress on goji berry micropropagation. The SVM model effectively predicted plant growth parameters under different stress conditions, contributing to the development of strategies for mitigating abiotic stress in plant tissue culture.

#### 2.1.6 Decision tree

A decision tree is used for both classification and regression. It models data using a tree-like structure where each internal node represents a decision based on a feature, branches represent outcomes of those decisions, and leaf nodes provide the final prediction (Thomas et al., 2020). The process starts from a root node that represents the entire dataset, and splits it into subsets based on selected features using specific splitting criteria: (i) Entropy (Information theory) measures the impurity or uncertainty in a dataset (Equation 8);

For a dataset *D* with *k* classes

(8) $$Entropy(D)=-\sum_{i=1}^{k}p_{i}log2\;p_{i}$$

Where:

p_i_ is the proportion of samples in class *i* in dataset *D.*

Entropy is 0 when all samples belong to one class (pure), and maximum when classes are equally distributed.

(ii) Information Gain (IG) that is used in ID3 algorithm, measures the reduction in entropy after a dataset is split on an attribute *A* (Equation 9);

(9) $$Gain(D,\;A)\;=\;Entropy\;(D)\;\frac{\left|Dv\right|}{\left|D\right|}\cdot \underset{v\;\in \text{ Values }(A)}{Entropry\;(Dv)}$$

Where:

*D* = dataset before split. Dv ​ = subset of *D* where attribute *A* has value v. $\frac{\left|Dv\right|}{\left|D\right|}\;$ = weight of the subset.

(iii) Gini impurity in the classification and regression trees (CART) algorithm, is another alternative to entropy to measure impurity of a dataset. Like entropy, Gini is 0 for pure datasets and it’s computationally simpler than entropy. These criteria assess how well a feature separates the data. Once the optimal feature is chosen, the dataset is partitioned based on that feature, and the process is repeated recursively on each subset until a stopping condition is met, such as reaching a maximum tree depth, or having a minimum number of samples per node. Since decision trees can overfit the training data, pruning techniques are employed to simplify the model. Common algorithms include iterative dichotomiser 3 (ID3) (using information gain), C4.5 (using gain ratio), CART (using gini impurity or MSE), and chi-squared automatic interaction detector (CHAID) (using Chi-square tests).

**Research application**

Aasim et al. (2023) successfully established an efficient *in vitro* regeneration protocol for *Sorghum bicolor* using direct organogenesis from mature zygotic embryo explants. The use of MS-medium supplemented with varying concentrations of BAP alone or in combination with IBA or NAA significantly influenced shoot count and shoot length, with optimal results observed for 2 mg/L BAP + 0.25 mg/L NAA and 2.0 mg/L BAP, respectively. Statistical analyses using factorial regression, pareto charts, and surface modeling confirmed the substantial effects of cytokinin–auxin interactions. Moreover, the integration of artificial intelligence-based models, including multilayer perceptron (MLP), random forest (RF), and XGBoost, demonstrated the superior predictive accuracy of MLP, highlighting the potential of AI in optimizing tissue culture protocols. In parallel, studies on wild apricot (*Prunus armeniaca*) (Kovalchuk et al., 2017) and hazelnut (*Corylus avellana*) employed decision tree algorithms such as CART, CHAID, and exhaustive CHAID to refine media composition and identify key mineral concentrations affecting shoot growth parameters. Particularly, CART proved effective in modeling complex nonlinear relationships and establishing specific cutoff points for components such as KH_2_PO_4_, MgSO_4_, and CuSO_4_. These findings underscore the relevance of decision tree-based modeling in deciphering multifactorial effects in plant tissue culture, offering a robust framework for media optimization across diverse species (Akin et al., 2017, 2018). The synergy between traditional statistical methods, AI models, and decision tree algorithms enhanced the ability to develop genotype-independent, efficient micropropagation systems, vital for crop improvement and biotechnological applications.

#### 2.1.7 Random forest

Random forest algorithm combines multiple decision trees to make more accurate predictions. Each decision tree in the random forest examines different random subsets of the data. The results from these trees are then combined via majority voting for classification (to determine the final class) or by averaging the predictions for regression (Breiman, 2001). This approach helps improve accuracy and reduce errors. Another key aspect of RF is the depth of the trees. In most cases, the individual decision trees are grown to their full depth without any pruning, making them very complex and potentially prone to overfitting if considered independently. However, this risk is mitigated in RF because the trees are aggregated, and their combined predictions help prevent overfitting. The two common ensemble techniques used in random forests to improve the performance of models are (i) bootstrap aggregating (bagging) is a core principle behind random forests, and it helps improve model accuracy and robustness. Samples are trained based on different decision trees. After training, the predictions from all the trees are aggregated: for classification, the final class is determined by majority voting (i.e., the class chosen by most trees), and for regression, the predictions are averaged.

Formula (Majority voting in random forest) (Equation 10):

(10) $$\hat{y}=arg\;max\left(\sum_{b=1}^{B}I\;\left(y_{b\;=}c\right)\right)$$

Where:

- *I* (y_b =_*c*)is an indicator function that returns 1 if y_b_=*c* and 0 otherwise,
- *B* is the number of trees in the forest.

(ii) boosting, on the other hand, focuses on reducing bias by sequentially training models. Each new tree in a boosting algorithm is trained to correct the mistakes made by the previous trees, with more attention given to misclassified data points. The final prediction is a weighted combination of the trees’ outputs, where trees with better performance have more influence. The other boosting techniques like gradient boosting machine (GBM), extreme gradient boosting machine (XGBM), LightGBM, and CatBoost become essential to confront the machine learning more deliverable with accuracy and precision.

**Research application**

*In vitro* propagation is an essential technique for conserving and mass-producing economically valuable plant species, yet it faces persistent challenges such as low germination rates, contamination, and genotype-specific responses. For instance, the *in vitro* germination of *Cannabis sativa*, historically difficult due to low germination and high contamination, was optimized using five ML models. Random Forest (RF) outperformed others with F1 scores between 0.98–1.00, identifying an optimal hydrogen peroxide concentration of ~2.2% (Aasim et al., 2022). Similarly, in aquatic plants like *Hemianthus callitrichoides*, RF and MLP models demonstrated high accuracy in predicting growth based on media composition (Özcan et al., 2023). In *Passiflora caerulea*, RF and GRNN, coupled with genetic algorithms (GA), successfully modeled shoot regeneration, identifying a precise hormonal combination for optimal results (Jafari and Daneshvar, 2023). Furthermore, in *Punica granatum*, RF and XGBoost, supported by novel tools like the global performance indicator (GPI) and NSGA-II, showed high fidelity in shoot proliferation predictions (Zarbakhsh et al., 2024). Finally, ML applications in drought-stressed *Fragaria × ananassa* highlighted genotype-specific modeling, with RF achieving the highest overall accuracy (Şimşek, 2024). Collectively, these examples underscore the versatility and predictive power of ML, particularly RF and other machine learning algorithms, in optimizing tissue culture protocols across diverse species.

### 2.2 Unsupervised learning

Unsupervised learning is a type of machine learning that works with unlabeled data. These algorithms are designed to identify patterns and relationships within the data on their own, without any prior knowledge of what the data represents (Nasrabadi, 2007). There are three primary types of unsupervised learning algorithms. (i) Clustering algorithms group data points based on similarity to uncover patterns without predefined labels. Common methods include K-means, which partitions data into *K*-clusters based on distance; hierarchical clustering, which creates a tree-like structure of nested clusters; and density-based clustering (DBSCAN) which identifies dense regions while treating sparse points as noise. Mean-shift clustering shifts points toward high-density areas to form clusters, while spectral clustering uses graph theory to group data based on relationships between points. (ii) Association rule learning identifies relationships between variables in large datasets, commonly used in market basket analysis to find items frequently purchased together. Key algorithms include Apriori, which iteratively finds frequent item sets, which improves efficiency by avoiding candidate generation; and Eclat, which uses set intersections. (iii) Dimensionality reduction techniques reduce the number of features in a dataset while preserving meaningful information, improving model simplicity, computational efficiency, and visualization. Principal component analysis (PCA) transforms data into uncorrelated components that capture maximum variance, while linear discriminant analysis (LDA) finds projections that enhance class separability (Tipping and Bishop, 1999; Hastie et al., 2009b) (**Figure 2B**).

**Research application**

A high-throughput phenotyping method was developed to efficiently collect trait data using imaging systems during key crop growth stages. To reduce dependence on human-labeled data for image-based trait extraction, KAT4IA is introduced, a self-supervised learning pipeline that applies K-means clustering to greenhouse images to automatically generate training data for field-based phenotyping (Guo et al., 2021). Metabolomics was integrated with transcriptomic analysis, to detect metabolic responses to combined stress in *Medicago truncatula*. LC-HRMS data from roots and leaves were analyzed using the gaussian process 2-sample test, K-means clustering, and affinity propagation for temporal clustering. Results revealed known stress markers, including altered sucrose and citric acid levels, with combined stress amplifying drought effects. While leaf responses were more pronounced, fusarium-related changes were also observed in roots (Dickinson et al., 2018).

### 2.3 Reinforcement learning

Reinforcement learning is a branch of machine learning where an agent learns to make decisions by interacting with an environment to maximize cumulative rewards over time. The agent observes the current state, takes actions, and receives feedback in the form of rewards, adjusting its strategy, or policy, to improve future outcomes (Szepesvári, 2010). A central challenge in RL is balancing exploration (trying new actions) and exploitation (leveraging known rewarding actions). This process is often modeled using markov decision processes (MDPs), which define states, actions, transition probabilities, and rewards. There are several categories of RL algorithms. Value-based methods, such as Q-learning and state-action-reward-state-action (SARSA), focus on estimating value functions that predict the expected future rewards of actions taken in given states (Watkins and Dayan, 1992). More recently, deep reinforcement learning leverages neural networks to handle complex, high-dimensional environments, with algorithms such as deep Q-networks (DQN) and proximal policy optimization (PPO) achieving state-of-the-art results. Overall, reinforcement learning provides a powerful framework for training agents to make sequential decisions in uncertain and dynamic environments (Hastie et al., 2009b) (**Figure 2C**).

**Research application**

The use of RL in plant biology and agriculture presents a promising avenue for addressing both fundamental biological questions and applied agricultural challenges. Across three distinct domains, plant organ development, crop breeding, and field management, RL has demonstrated its versatility and effectiveness as a decision-making and optimization framework and yet be expanded in the field of plant tissue culture. The reinforcement learning was used to model the biomechanics of the “searcher shoot”, a plant organ specialized for spatial exploration. By framing mass distribution and structural constraints as a markov decision process (MDP), the authors created the *Searcher-Shoot* environment to simulate adaptive growth strategies. Results showed consistent shoot tapering, suggesting that plants may naturally adopt efficient mass allocation to optimize elongation without surpassing stress limits. The close match between simulated and empirical data highlights the potential of RL to model the adaptive complexity of plant morphology (Nasti et al., 2024). RL was applied to crop breeding, a domain challenged by slow generation turnover, high-dimensional decision spaces, and increasing environmental pressures due to climate change. By introducing a suite of Gym environments tailored for breeding simulations, the authors trained RL agents to make selection and crossing decisions based on real-world genomic data (Younis et al., 2024). In a parallel application, Balderas et al. (2025) examined RL’s potential in optimizing crop production management. Using the *gym-DSSAT* environment, a well-established crop simulation framework, they evaluated two widely used RL algorithms, proximal policy optimization (PPO) and deep Q-networks (DQN), across key agricultural tasks: fertilization, irrigation, and integrated management. Their findings revealed that PPO generally performed better in single-task settings (fertilization and irrigation), whereas DQN excelled in the mixed management task.

### 2.4 Semi-supervised learning

Semi-supervised learning is a hybrid approach that lies between supervised and unsupervised learning, combining both labeled and unlabeled data. This method is particularly useful when labeled data is scarce or expensive to obtain, but a large amount of unlabeled data is available, helping to improve model performance through various strategies (Zhu and Goldberg, 2009). Self-training iteratively labels unlabeled data using an initial model trained on labeled data. Co-training leverages multiple classifiers, each trained on different data views, to label unlabeled examples. Generative models, like gaussian mixture models (GMMs) and hidden markov models (HMMs) predict labels based on learned data distributions (Hastie et al., 2009b) (**Figure 2D**).

**Research application**

Pure self-supervised learning (SSL) methods, like FixMatch and others, haven’t been widely adopted in plant tissue culture research. This is largely because of the small dataset sizes and the challenges in reliably generating pseudo-labels for *in-vitro* outcomes. Instead, most recent studies have focused on label-efficient models, approaches that combine regression or classification techniques with limited experimental data or utilize self- and semi-controlled setups. A semi-supervised framework, DM_CorrMatch, was proposed for rapeseed inflorescence segmentation. It integrates data augmentation with a denoising diffusion probabilistic model (DDPM) to address limited annotated data. The Mamba-Deeplabv3+ network used in the study captures both local and global features, enhancing segmentation accuracy despite complex backgrounds and variable inflorescence poses. Validated on the rapeseed flower segmentation dataset (RFSD), the model achieved an intersection over union (IoU) of 0.886, precision of 0.942, and recall of 0.940 (Li et al., 2025). A study focused on plant leaf disease recognition using semi-supervised few-shot learning approach was carried out to address the challenge of limited labeled data in plant pathology. By combining source and target domains from the PlantVillage dataset, the iterative semi-supervised method achieved an accuracy improvement of up to 4.6% (Li and Chao, 2021). Najafian et al. (2023) explored the use of deep learning for semantic segmentation in agriculture with minimal annotation. By applying domain adaptation techniques, the authors achieved impressive segmentation results for wheat heads with just two annotated images, demonstrating the power of synthesized datasets and the effectiveness of using limited labeled data.

## 3 Artificial neural networks in plant tissue culture

**Figure 3:**
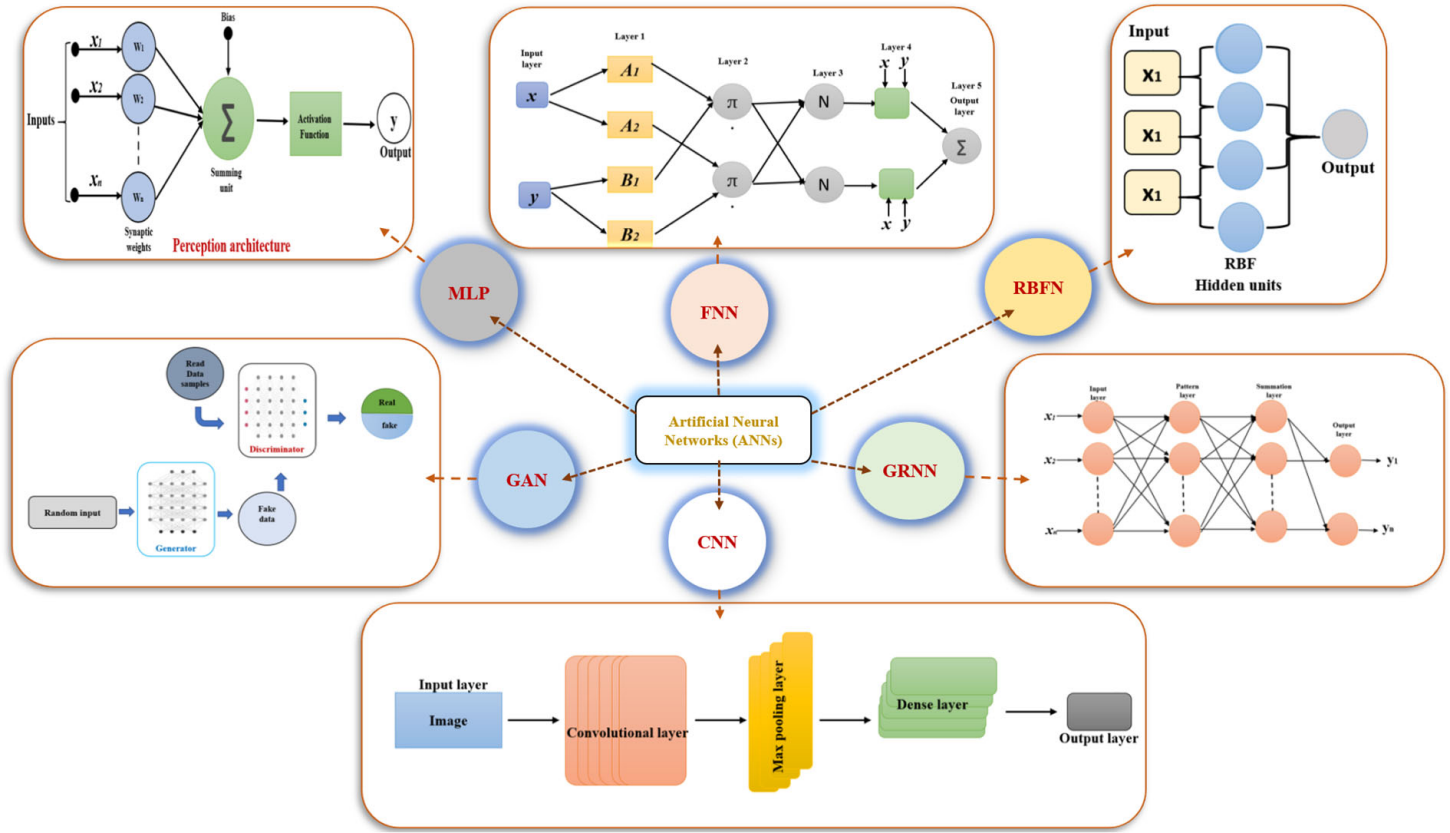
Architectural variants of artificial neural networks (ANNs). MLP, Multilayer Perceptron; FNN, Fuzzy Neural Network; RBFN, Radial Basis Function Network; GAN, Generative Adversarial Network; CNN, Convolutional Neural Network; GRNN, General Regression Neural Network.

Artificial neural networks have been extensively applied in plant tissue culture to predict, analyze, and optimize processes including plant growth, tissue regeneration, callus formation, and disease detection (Prasad and Gupta, 2008) (**Table 2**). Several types of ANNs can be employed for different purposes in plant tissue culture research (**Figure 3**). Some of the most common types include.

**Table 2:** Applications of artificial neural networks (ANNs) approaches/models used in plant tissue culture and other prediction studies.

Algorithm/Model	Hybrid models	Plant species	Application/Prediction output	Performance metric values	Reference
Multi-Layer perception (MLP)	MLP + SVR	*Chrysanthemum morifolium*	Optimizing somatic embryogenesis	R^2^: 0.99 SVM, 0.91 MLP; RMSE: 0.94 SVM, 2.07 MLP	Hesami et al., 2020a
	MLP + SPDGA	*Daucus carota*	Optimization callus induction	R^2^: 0.95 MLP, 0.83 RBF; RMSE: 0.451 MLP, 0.480 RBF	Fallah Ziarani et al., 2022
	MLP + NSGA-II	*Erysimum cheiri*	Predict shoot number, shoot length, and callus weight	R^2^: 0.84 MLP; RMSE: 0.68 MLP	Fakhrzad et al., 2022
	MLP + GRNN-GA	*Passiflora caerulea*	*In vitro* rooting responses	R^2^: 0.99 GRNN, 0.96 RF; RMSE: 3.08 GRNN, 3.12 RF	Jafari and Daneshvar, 2023
	MLP + MLPNN + KNN + GEP	*Juglans regia L.*	*In vitro* proliferation	R^2^: 0.428 MLR, 0.672 KNN; RMSE: 3.313 MLR, 1.756 KNN	Sadat-Hosseini et al., 2022
	MLP	*Andrographis paniculata*	Prediction of secondary metabolite enhancement	R^2^: 0.9716 MLP; RMSE: 0.18 MLP; Accuracy: 90%	Champati et al., 2022
	MLP + RF + RBF + XBoost	*Fragaria × ananassa*	Prediction of plant height and proliferation	R^2^: 0.55 MLP, 0.59 SVM, 0.78 RF; RMSE: 0.91 MLP, 0.76 SVM, 0.57 RF	Şimşek, 2024
Radial Basis Function Networks (RBFN)	RBF + MLP	*Lamiaceae members*	Optimization of *in vitro* sterilization	R^2^: 0.68 RBF; RMSE: 7.42 RBF	Ivashchuk et al., 2018
	RBF + NSGA-II	*Chrysanthemum morifolium*	Shoot proliferation	R^2^: 0.88 RBF; RMSE: 13.38 RBF; Accuracy: 98.5%	Hesami et al., 2019
	RBF + MLP + GRNN	*Petunia* spp.	Optimization of callogenesis	R^2^: 0.801 MLP, 0.837 GRNN, 0.811 RBF; RMSE: 9.525 MLP, 7.178 GRNN, 9.131 RBF	Rezaei et al., 2023a
Generalized Regression Neural Network (GRNN)	GRNN + GA	*Triticum* spp.	Shoot regeneration	R^2^: 0.78 GRNN; RMSE: 14.76 GRNN	Hesami et al., 2020c
	GRNN + FOA	*Corylus avellana*	Cell suspension culture	R^2^: 0.90 GRNN; RMSE: 16.93 GRNN	Salehi et al., 2021
	GRNN + RBF + MLP	*Petunia* spp.	*In vitro* seed sterilization	R^2^: 0.844 MLP, 0.886 GRNN, 0.863 RBF; RMSE: 20.645 MLP, 14.836 GRNN, 18.370 RBF	Rezaei et al., 2023b
Fuzzy Neural Networks (FNN)	Neuro-fuzzy logic (IF-THEN rule set)	*Vitis vinifera*	Microshoot rooting	R^2^: 0.940; *f*-ratio: 346.0	Gago et al., 2010
	Neuro-fuzzy logic (IF-THEN rule set)	*Prunus* spp.	Media formulation	R^2^: 91.38; *f*-ratio: 6.77	Gago et al., 2011
	Neuro-fuzzy logic (IF-THEN rule set)	*Prunus* spp. *(GF677)*	Micropropagation & media formulation	R^2^: 77.48; *f*-ratio: 15.73	Alanagh et al., 2014
	Neuro-fuzzy logic + GA	*Pistacia vera*	Micropropagation & media formulation	R^2^: 84.84; *f*-ratio: 76.54	Alanagh et al., 2017
	Neuro-fuzzy logic	*Actinidia arguta*	*In vitro* proliferation	R^2^: 89.495; *f*-ratio: 9.030	Hameg et al., 2020
	ANFIS + NSGAII	*Chrysanthemum morifolium*	Somatic embryogenesis	R^2^: 0.88 RBF; RMSE: 13.38 RBF	Hesami et al., 2019
	ANFIS + GA	*Corylus avellana*	Cell suspension cultures	R^2^: 0.88 ANFIS; RMSE: 16.68 ANFIS	Farhadi et al., 2020
Generative Adversarial Networks (GAN)	WacGAN	Multiple species of plant seedlings	Accurate plant identification	Recognition accuracy: 86.2%	Simonyan and Zisserman, 2014
	Spe-GAN & Spa-GAN	*Gossypium* spp.	Early-stage detection of Verticillium wilt	Accuracy: 96.3%; Precision: 95.6%	Tan et al., 2025
Convolutional Neural networks (CNN)	CNN + IoT	*Cocos nucifera*	Disease classification and characterizing tissue culture calli	mAP50: 0.865; Accuracy: 88%	Shavindi et al., 2024
	YOLOv8 + CNN	*Cocos nucifera*	Plant growth monitoring and browning disease severity identification	Precision: 0.925; mAP50: 0.884	Rajapaksha et al., 2024
	YOLOv8 + CNN	*Cocos nucifera*	Detecting and quantifying infections	Accuracy: 93%	Heenkenda et al., 2024

R2, Coefficient of determination; RMSE, Root mean square error; MLP, Multi-Layer perception; SVR, Support Vector Regression; SPDGA, Self-adaptive Population Differential Genetic Algorithm; NSGA-II, Non-dominated Sorting Genetic Algorithm II; GRNN-GA, Generalized Regression Neural Network with Genetic Algorithm; MLPNN, Multi-Layer Perceptron Neural Network; RBF, Radial Basis Function; RF, Random Forest; FOA, Fruit fly algorithm; ANFIS, Adaptive Neuro-Fuzzy Inference System; WacGAN, Wasserstein auxiliary classifier GAN; Spe-GAN, Spectral enhancement GAN; Spa-GAN, Spatial enhancement GAN; mAP, Mean average precision.

### 3.1 Multi-layer perception

A Multi-layer perceptron is consisting of fully connected layers that transform input data through various dimensions. It is called “multi-layer” because it includes an input layer, one or more hidden layers, and an output layer. The input layer contains neurons representing each feature in the data, while the hidden layers process the information passed from the input. The number of hidden layers and neurons in each can vary. Finally, the output layer generates the prediction or result, with a number of neurons corresponding to the number of outputs. The main purpose of an MLP is to model complex relationships between inputs and outputs (Christopher, 1995). Each neuron in the hidden layers processes the input by first calculating a weighted sum of the inputs, given by the formula (Equation 11).

(11) $$z=\sum_{i}w_{i}x_{i}+b$$

Where *xi*​​ is the input feature, *wi*​​ is the corresponding weight and b is the bias term. This weighted sum z, is then passed through an activation function to introduce non-linearity. Once the network generates an output, the next step is to compute the loss using a loss function, which compares the predicted output to the actual label. For a classification task, the binary cross-entropy loss function is typically used (Equation 12):

(12) $$L=-\frac{1}{N}\left[\sum_{i=1}^{N}\left[y_{i}log({\hat{y}}_{i})+(1-y_{i})log(1-{\hat{y}}_{i}\right)\right]$$

where *y_i_* is the actual label, ${\hat{y}}_{i}$ is the predicted label, and N is the number of samples.

For regression problems, mean squared error (MSE) is often used (Equation 13):

(13) $$MSE(t)=\frac{1}{|t|}{\sum_{i\in \;t}\left(yi–\hat{y}t\right)}^{2}$$

Where $\hat{y}\text{t}$ is the average label value at node *t*, ∣t∣ is the number of samples at node *t*.

The goal of training an MLP is to minimize the loss function by adjusting the network’s weights and biases through backpropagation. MLPs use optimization algorithms to iteratively update weights and biases during training. Stochastic Gradient Descent (SGD) updates weights using individual samples or small batches, while the Adam optimizer enhances SGD by incorporating momentum and adaptive learning rates, enabling more efficient and effective training through dynamic adjustment of learning rates (Osama et al., 2015).

**Research application**

The critical role of optimizing somatic embryogenesis for successful gene transformation in chrysanthemum was analyzed by comparing two ML models, multilayer perceptron (MLP) and support vector regression (SVR). The study found that SVR outperformed MLP, achieving a higher predictive accuracy (R² > 0.92 vs. R² > 0.82). When combined with the NSGA-II optimization algorithm, the SVR model led to exceptional results including 99.09% embryogenesis efficiency and an average of 56.24 embryos per explant. This study highlighted the potential of integrating machine learning with evolutionary algorithms to enhance plant tissue culture outcomes (Hesami and Jones, 2020b). Similarly, Fallah Ziarani et al. (2022) employed an MLP model integrated with a single point discrete genetic algorithm (SPDGA) to optimize callus induction in carrots. The MLP model outperformed the radial basis function (RBF) network, achieving R² values around 0.95. Sensitivity analysis revealed MS-salt concentration as the most influential factor, underscoring the value of targeted ML optimization in improving regeneration protocols. In *Erysimum cheiri*, a lesser-studied medicinal plant, Fakhrzad et al. (2022) developed MLP-based models to predict shoot number, shoot length, and callus weight, with strong predictive performance (R²=0.84–0.99). Optimization using NSGA-II helped fine-tune hormone concentrations, further validating the reliability of the model. A focused study on rooting in *Passiflora caerulea*, was carried out by applying a hybrid general regression neural network–genetic algorithm (GRNN-GA) model (Jafari and Daneshvar, 2023). This study demonstrated the GRNN-GA model’s effectiveness in capturing complex *in vitro* rooting responses. GRNN achieved excellent accuracy (R² > 0.92), and the optimization process produced favorable rooting outcomes based on auxin types and explant sources. The application of MLP-based artificial neural network was successful to correlate soil nutrient levels with andrographolide content in *Andrographis paniculata* across 150 accessions in eastern India (Champati et al., 2022). Their best-performing model (14-12–1 architecture) achieved a high accuracy (R² = 0.9716), and the optimization process raised andrographolide content from 3.38% to 4.90%. This study showcased the broader utility of ML in site selection and secondary metabolite enhancement, extending its value beyond traditional tissue culture. Sadat-Hosseini et al. (2022) compared multiple ML models, including MLPNN, K-nearest neighbors (KNN), and gene expression programming (GEP), against traditional multiple linear regression (MLR) for predicting *in vitro* proliferation in persian walnut (*Juglans regia*). GEP, particularly when optimized with particle swarm optimization (PSO), provided the most accurate predictions (R² = 0.802 for the ‘chandler’ cultivar, significantly outperforming MLR (R² = 0.412). The study also validated KNN as a simple yet effective tool, highlighting the range of viable ML approaches depending on data complexity and application needs. Şimşek (2024) extended ML applications to genotype-specific optimization in lavender and strawberry cultivars. In lavender, random forest (RF) outperformed other models (MLP, RBF, XGBoost, and Gaussian process) in predicting root traits, particularly for the ‘Festival’ and ‘Fortuna’ genotypes. Meanwhile, MLP and GP models were more effective in predicting traits for ‘Rubygem’. Similarly, in strawberries under PEG-induced drought stress, RF achieved the highest accuracy (91.16%) for root trait prediction, while MLP and GP better predicted plant height and proliferation. These findings emphasize the need to tailor model selection to genotype and trait specificity.

### 3.2 Radial basis function networks

Radial basis function network is designed primarily for tasks such as function approximation, classification, and regression. This is particularly effective for problems where the relationship between input and output is nonlinear. At their core, RBFN is a form of feedforward neural network consisting of three layers: the input layer, which receives the input features; the hidden layer, which contains radial basis function neurons that transform the input into a higher-dimensional space; and the output layer, which generates the final output after the transformation (Lin et al., 2020a). A radial basis function is a real-valued function whose output depends only on the distance between the input and a specific point (often referred to as a center). The output of the function is maximum at the center and decreases as the input moves farther away from it.

The output of the network can be expressed as (Equation 14):

(14) $$y\left(x\right)=\sum_{i=1}^{N}w_{i}\phi \left(∥x-c_{i}∥,\sigma_{i}\right)\;$$

Where:

*y(x)* the output of the network, *w_i_​* are the weights between the hidden neurons and the output layer, ϕ(∥x−c_i_∥, σ_i_) is the output of the radial basis function at neuron *i*, c_i_ ​ is the center of the *i*^th^ radial basis function, σ_i_ is the spread (or width) of the *i*^th^ radial basis function.

Commonly, optimization algorithms like gradient descent, least squares, or LMS (Least mean squares) are used to minimize the error between the predicted and actual outputs.

**Research application**

Ivashchuk et al. (2018) applied neural networks to model and optimize the sterilization stage for plant seeds, specifically targeting rare and medicinal plants. The study used both MLP and RBF networks to predict the optimal sterilization parameters (sterilant type, concentration, and exposure time). The combination of radial basis function (RBF) networks and the non-dominated sorting genetic algorithm-II (NSGA-II) was used to model and optimize the medium compositions for shoot proliferation in chrysanthemum (Hesami et al., 2019). The study is focused on four outputs, proliferation rate (PR), shoot number (SN), shoot length (SL), and basal callus weight (BCW), based on the concentrations of four variables: BAP, IBA, phloroglucinol (PG), and sucrose. The results showed high prediction accuracy with R² values of 0.88, 0.91, 0.97, and 0.76 for PR, SN, SL, and BCW, respectively. Importantly, the predicted and actual outcomes showed negligible differences, validating RBF-NSGAII as a reliable and efficient tool for optimizing *in vitro* organogenesis. Rezaei et al. (2023a) focused on the optimization of callogenesis in petunia by developing a predictive model using three machine learning algorithms: multilayer perceptron (MLP), radial basis function (RBF), and generalized regression neural network (GRNN). The goal was to optimize the concentrations of phytohormones to enhance callus formation rate (CFR) and callus fresh weight (CFW). Among the models, GRNN outperformed MLP and RBF with R² values of ≥83%. The study also employed sensitivity analysis, which revealed that IBA was the most influential phytohormone for callogenesis. By integrating GRNN with a genetic algorithm (GA), the study identified an optimized set of phytohormone concentrations that maximized CFR to 95.83%. Experimental validation confirmed the accuracy of the predicted optimal conditions, showing no significant difference between the experimental and GA-predicted results. This approach demonstrates the successful integration of machine learning, sensitivity analysis, and genetic algorithms to optimize tissue culture conditions in a controlled and efficient manner.

### 3.3 Generalized regression neural network

The generalized regression neural network is a special form of radial basis function (RBF) networks and functions as a non-parametric, memory-based model that provides a smooth approximation of the underlying relationship between inputs and outputs. One of its main advantages is its ability to approximate any arbitrary function directly from training data without requiring an assumed functional form. GRNN consists of four layers: the input layer, which contains neurons representing the features of the input vector and simply passes the data forward without processing; the pattern layer (or radial basis layer), where each neuron corresponds to a training sample and calculates the euclidean distance between the input and that sample, then applies a gaussian kernel to convert this distance into a similarity measure (Equation 15).

(15) $$K_{i}=exp\left(-\frac{{\left(x-x_{i}\right)}^{T}\left(x-x_{i}\right)}{2\sigma^{2}\;}\right)$$

Where *x* is input vector, x*_i_* is training sample vector and σ is smoothing parameter (spread or bandwidth).

This structure allows GRNN to provide smooth and effective regression estimates directly from the data, making it a powerful tool for nonlinear function approximation (Specht, 1991).

**Research application**

Hesami et al. (2020c) used GRNN-GA to model shoot regeneration in wheat, addressing genotype-dependent variability. Based on 10 input variables, GRNN-GA achieved good predictive performance (R² = 0.78) and identified 2,4-D, explant type, and genotype as key factors, supporting more efficient, genotype-independent regeneration protocols. A cost-effective method for paclitaxel production in *Corylus avellana* was demonstrated using cell suspension culture (CSC) enhanced by fungal elicitors (Salehi et al., 2021). In this study, a general regression neural network optimized by the fruit fly algorithm (GRNN-FOA) was applied to predict and optimize paclitaxel biosynthesis and biomass production, using four input variables: cell extract (CE), culture filtrate (CF), elicitor adding day, and harvesting time. GRNN-FOA showed higher accuracy (R² = 0.88–0.97) than traditional regression (R² = 0.57–0.86), and performed comparably to MLP-GA, with slight advantages for MLP-GA in total and extracellular paclitaxel prediction. GRNN-FOA optimization predicted a maximum paclitaxel yield of 372.89 µg L^-^¹ under specific conditions, closely matching observed values, validating its potential in optimizing secondary metabolite production *in vitro.*Rezaei et al. (2023b) evaluated six disinfectants and immersion times on *Petunia* seed sterilization and germination. Among MLP, RBF, and GRNN models, GRNN performed best. They applied NSGA-II for multi-objective optimization, demonstrating that GRNN-NSGA-II effectively balances contamination control and germination success, offering a robust tool for plant tissue culture optimization.

### 3.4 Fuzzy neural networks

Neuro-fuzzy logic is a hybrid system that integrates neural networks with fuzzy logic principles to solve complex problems involving uncertainty and imprecision. By combining the learning capabilities of neural networks with the reasoning power of fuzzy logic, it proves especially effective in fields such as control systems, pattern recognition, decision-making, and adaptive systems (Ishibuchi, 1996). Unlike classical logic, where variables are strictly true or false, fuzzy logic allows for a continuum of truth values between 0 and 1. This approach enables systems to model fuzzy rules while simultaneously learning from data. The system operates through four main steps (i) *fuzzyfication*, where input data is transformed into fuzzy values using membership functions (ii) *rule formation*, where fuzzy rules describe input-output relationships and can adapt automatically during learning (iii) *adaptive learning*, where the neural network adjusts the fuzzy rule parameters, like membership functions and rule weights, using algorithms such as backpropagation to minimize errors; and finally, (iv) *defuzzification*, where fuzzy outputs are converted into crisp values through methods like the centroid technique. Various neuro-fuzzy system implementations exist, notably the Adaptive Neuro-Fuzzy Inference System (ANFIS), known for its efficiency in function approximation. Another approach, the Fuzzy Neural Network (FNN), combines fuzzy logic with neural networks and often uses Mamdani-type rules, which enhance interpretability but increase computational cost. Additionally, Fuzzy C-Means, to partition the input space into clusters, enabling more effective decision-making by training neural networks on these fuzzy clusters (Talpur et al., 2023).

**Research application**

Recent advances in plant tissue culture optimization increasingly utilize AI, particularly neuro-fuzzy systems and hybrid models combining with evolutionary algorithms, to model complex relationships and enhance culture protocols. Initially, Gago et al. (2010) applied neuro-fuzzy logic to grapevine microshoot rooting, uncovering interactions, such as auxin-sucrose effects, not previously identified by statistical methods. This deepened the understanding of the micropropagation process and demonstrated the rapid applicability of AI modeling. The effective application of neuro-fuzzy logic to mine apricot micropropagation databases has revealed meaningful IF-THEN rules linking cultivars, mineral nutrients, and plant growth regulators with growth parameters (Gago et al., 2011). Their approach validated and extended traditional statistical findings by generating interpretable, reusable knowledge that supports future media optimization. In line with these applications, Alanagh et al. (2014) employed neuro-fuzzy logic to model macronutrient effects on GF677 peach × almond rootstock micropropagation. Their model pinpointed key ion interactions, such as NO3− × Ca2+, that significantly affect shoot quality and development, providing a powerful tool to infer optimal nutrient combinations and mitigate physiological disorders like hyperhydricity. Neuro-fuzzy logic and hybrid AI techniques to dissect nutrient and growth regulator influences on pistachio (*Pistacia vera*) micropropagation was given by Alanagh et al. (2017). By reducing complex media component combinations via design of experiments (DOE) and applying AI modeling, they uncovered critical ion interactions affecting shoot proliferation, quality, and physiological disorders, enabling more rational design of culture media. Hameg et al. (2020) applied neurofuzzy logic to kiwi (*Actinidia arguta*) micropropagation, revealing that BAP concentration predominantly influences shoot number, while a combination of BAP and GA_3_ affects shoot length. Their model also underscored the importance of the number of subcultures and media composition, highlighting AI’s role in interpreting multifactorial effects and guiding protocol refinement. Similarly, Hesami et al. (2019) introduced a hybrid adaptive neuro-fuzzy inference system combined with the non-dominated sorting genetic algorithm-II (ANFIS-NSGAII) for modeling somatic embryogenesis in chrysanthemum. Their results showed high predictive accuracy (R² > 92%) and identified optimal media compositions and light conditions to maximize embryogenesis frequency and somatic embryo number. Sensitivity analysis further highlighted 2,4-D as a critical factor, illustrating how hybrid AI models can guide precise optimization in complex biological systems. Farhadi et al. (2020) modeled paclitaxel production in *Corylus avellana* cell suspension cultures using ANFIS combined with genetic algorithms. Their model outperformed traditional regression approaches and successfully predicted optimal elicitor and methyl-β-cyclodextrin concentrations and timing to maximize paclitaxel yield, showcasing AI’s utility in bioproduct optimization.

### 3.5 Generative adversarial networks

Generative adversarial networks are a class of machine learning frameworks designed to generate new data instances that resemble a given dataset. They consist of two neural networks, the generator and the discriminator, that are trained simultaneously in a competitive setting. The unique aspect of GANs lies in their adversarial nature, where the generator creates data, and the discriminator tries to distinguish between real and fake data. Over time, as these networks “battle,” the generator learns to produce increasingly convincing data, while the discriminator improves its ability to differentiate between real and fake samples. As this adversarial process continues, both networks improve iteratively, with the generator producing more realistic data and the discriminator becoming more adept at distinguishing the two (Goodfellow et al., 2020).

**Research application**

Madsen et al. (2019) addressed the challenge of high intra-class variance and low inter-class variance in plant seedlings, an issue that hinders accurate plant identification using deep learning. To mitigate limited training data, they employed generative adversarial networks (GANs) to generate synthetic images across nine plant species. While the GAN-augmented model reached high recognition accuracy, misclassifications arose mainly during the dicotyledonous growth stage, where visual similarities between species and subtle shape differences confused the model. Notably, the synthetic data proved effective for pretraining a classification model, which performed well even before fine-tuning. Further fine-tuning with real data yielded only marginal improvement, indicating the robustness of the pretrained model. The early detection challenges of verticillium wilt (VW) in cotton, was addressed as a major concern for cotton yields globally (Tan et al., 2025). The study proposed an innovative method integrating GANs with hyperspectral imaging to enhance early-stage detection. Two models, Spe-GAN (spectral enhancement) and Spa-GAN (spatial enhancement), were developed to capture subtle symptoms of VW, a task complicated by limited data. The results showed that these GAN-based models significantly outperformed traditional machine learning (RF, SVM) and deep learning methods (LSTM, ResNet18), with Spe-GAN achieving 94.52% accuracy and Spa-GAN 91.78%. The approach’s success was largely attributed to its ability to augment limited data and enhance model interpretability, offering a new approach for early disease detection in cotton and potentially other plants.

### 3.6 Convolutional neural networks

A convolutional neural network is designed specifically for processing grid-like data, such as 2D images. Unlike fully connected neural networks (FCNs), CNNs use local receptive fields and shared weights to extract spatial hierarchies of features through convolutions. The input layer, is typically a 3D tensor (height × width × channels). Convolutional Layer, applies a number of filters (kernels) that scan across the input image to extract feature maps (Simonyan and Zisserman, 2014). A filter is a small matrix that slides over the input, performing element-wise multiplication and summing the results to form a single output value per position. Parameters include stride (the number of pixels the filter moves across the image) and Padding (Adding zero borders to control the spatial size of the output). In CNNs, pooling layers perform down sampling to reduce the spatial dimensions of feature maps, lowering computational complexity and helping to prevent overfitting. Max pooling is the most common method, selecting the maximum value within a local window to preserve dominant features. After several convolution and pooling stages, the network transitions to fully connected layers, where flattened feature maps are used for high-level reasoning. The final output layer, also fully connected, uses activation functions suited to the task, softmax for multi-class classification and sigmoid for binary or multilabel classification.

**Research application**

The integration of deep learning, image processing, and IoT technologies in coconut tissue culture monitoring, as demonstrated by Shavindi et al. (2024), presents a transformative shift in agricultural biotechnology. By automating the classification of callus tissues and enabling continuous culture monitoring, the study addresses critical inefficiencies in traditional tissue culture methods. Complementing this work, Rajapaksha et al. (2024) focused on automating the measurement of plant growth parameters using deep learning models, particularly YOLOv8 and CNN architectures. Their research achieved high accuracy in tasks such as flask detection (precision: 0.990, mAP@50: 0.995), leaf and stem segmentation, and root classification. Moreover, InceptionV3 achieved 96% accuracy in browning disease classification, demonstrating the potential of CNNs in plant health diagnostics. Heenkenda et al. (2024) further highlighted the limitations of conventional tissue culture techniques, especially in early disease detection. Their proposed machine learning-based approach for identifying and predicting bacterial and fungal infections through image analysis offers a critical advancement. The integration of technological tools with agronomical expertise bridges the gap between traditional farming practices and modern precision agriculture, fostering sustainable cultivation and timely disease management.

## 4 Advancements in genome editing technologies

Genome editing is a revolutionary technology that enables precise modifications to an organism’s DNA, including insertions, deletions, and replacements, to modify biological traits. Several genome editing tools have been developed, each with distinct mechanisms and limitations. Meganucleases are natural endodeoxyribonucleases that recognize and cleave DNA sequences typically 20–30 base pairs long. Although their recognition sites can be re-engineered for site-specific editing, the structural complexity and time-consuming modification process have limited their widespread use (Ashworth et al., 2006; Grizot et al., 2009). Zinc Finger Nucleases (ZFNs) combine engineered zinc finger proteins with the FokI endonuclease to generate site-specific DNA breaks (Kim et al., 1996), while Transcription Activator-Like Effector Nucleases (TALENs) use TALE-derived DNA-binding domains and FokI to induce targeted double-stranded breaks (DSBs) (Christian et al., 2010). Despite their proven efficacy, the intricate and labor-intensive design and assembly of meganucleases, ZFNs, and TALENs have restricted their adoption.

**Figure 4:**
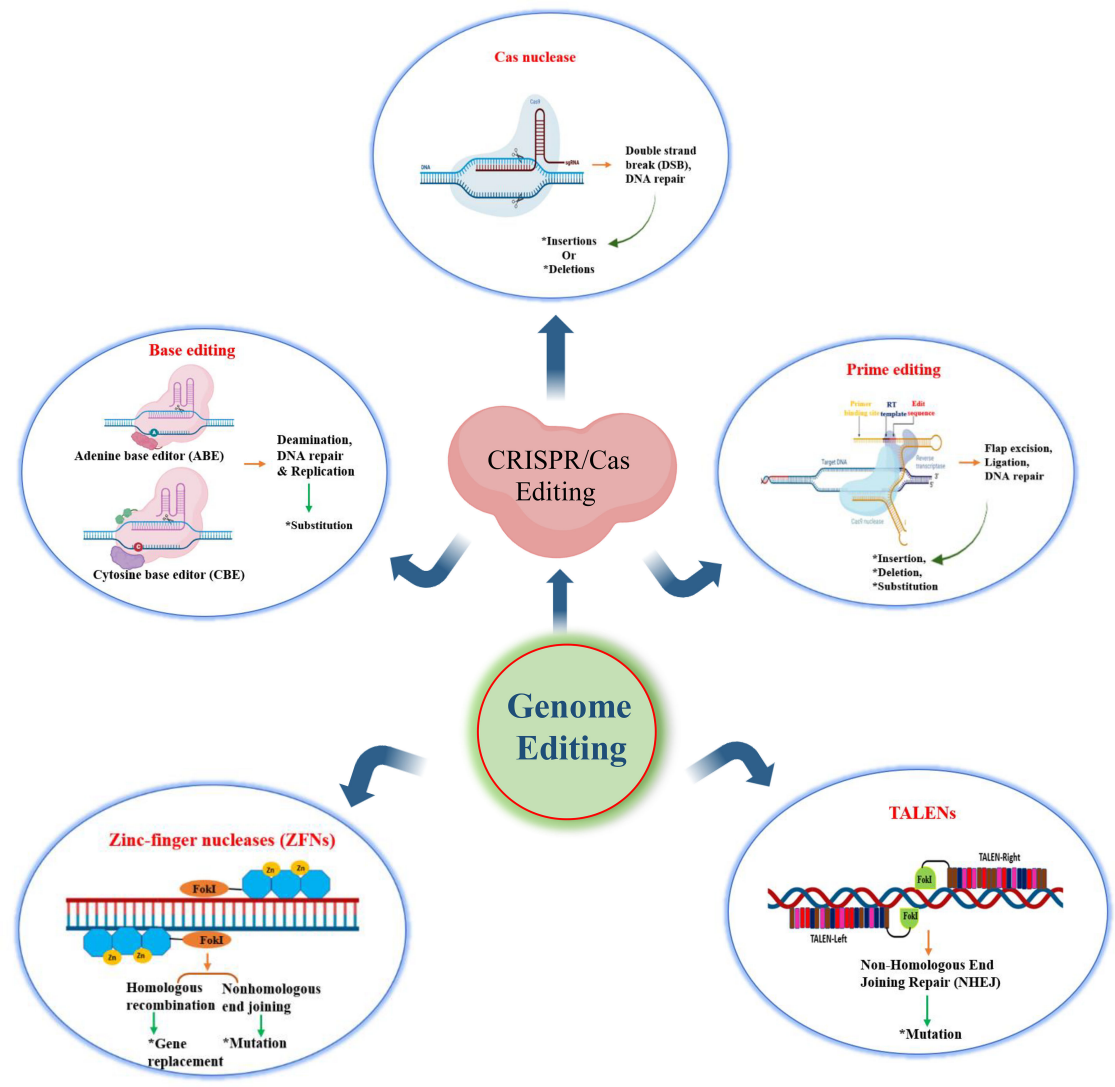
Overview of genome editing techniques: CRISPR/Cas and other nuclease-based approaches (This figure was drawn using free version of BioRender; https://www.biorender.com).

In contrast, CRISPR/Cas systems (Makarova et al., 2011), particularly CRISPR/Cas9 and CRISPR/Cas12a, have gained prominence due to their simplicity, flexibility, and efficiency. Originating as an adaptive immune system in bacteria and archaea, CRISPR/Cas uses guide RNAs to direct Cas proteins to specific DNA sequences adjacent to a PAM (protospacer adjacent motif), enabling targeted cleavage (Garneau et al., 2010). CRISPR/Cas systems introduce DSBs, which are repaired by either non-homologous end joining (NHEJ), an error-prone process leading to insertions or deletions or homology-directed repair (HDR), which allows precise gene correction using a template. Early CRISPR studies in plants demonstrated successful gene knockouts and modifications, highlighting its powerful potential in plant biology (Feng et al., 2013; Li et al., 2013; Nekrasov et al., 2013). Recent advancements include base editing and prime editing. Base editors combine a Cas9 nickase or dead Cas9 (dCas9) with a deaminase enzyme to convert specific nucleotides without inducing DSBs (Komor et al., 2016). Prime editors fuse dCas9 with reverse transcriptase and use a prime editing guide RNA (pegRNA) to direct edits at a target site through a templated reverse transcription mechanism (Anzalone et al., 2019). These next-generation tools have been successfully applied across various plant species, significantly advancing precision breeding and crop improvement (Lin et al., 2020b) (**Figure 4**).

### 4.1 Searching and screening strategy

The identification phase involves constructing search queries using combinations of keywords, that include: *“CRISPR EDITING USING MACHINE LEARNING”, “DEEP LEARNING AND CRISPR EDITING”, “MACHINE AND DEEP LEARNING AND CRISPR EDITING”*. This study draws from multiple online databases like pubmed central, scopus and google scholar, compiling research on the application of machine learning (ML) and deep learning (DL) in genome editing technologies. The articles were selected based on their relevance and uniqueness. Peer-reviewed research papers and review articles were considered for analysis. Initial screening was conducted at the abstract level, followed by data extraction and full-text analysis.

### 4.2 Role of machine learning/deep learning in CRISPR-Cas9 genome editing

The accuracy and accessibility of genome-editing tools particularly CRISPR-associated Cas9 protein has revolutionized the biological research and therapeutic development. A key objective is improving guide RNA (gRNA) design to enhance on-target efficiency (OTE) and minimize off-target effects (OFTE). This review explores recent advances in computational methods, particularly ML and DL, for predicting gRNA performance. It also highlights the role of AI in advancing base editing, prime editing and epigenome editing as well as discusses about tools for predicting gene editing outcome’s and optimizing editing proteins.

#### 4.2.1 sgRNA-DNA sequence encoding

**Figure 5:**
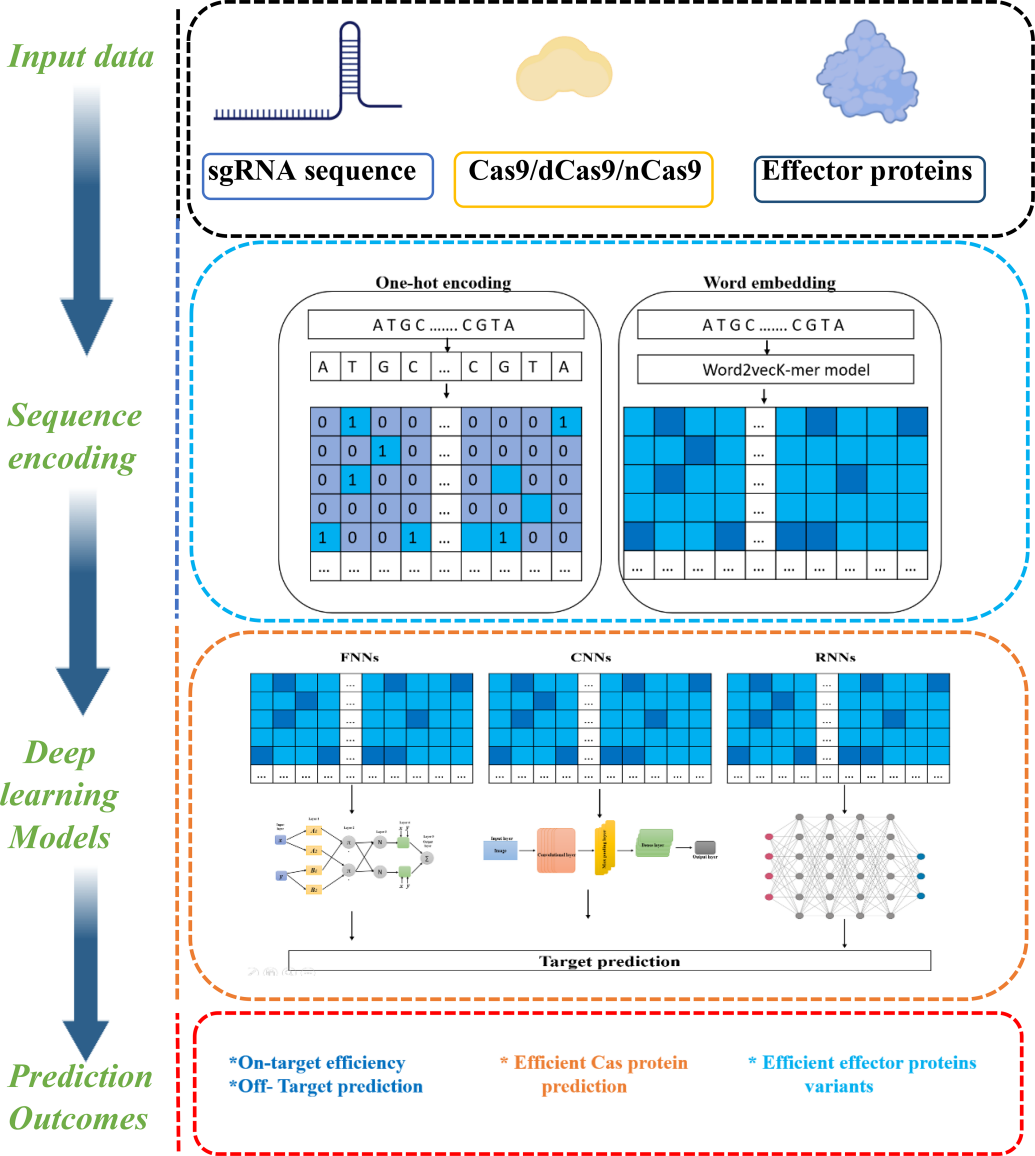
Schematics of deep learning framework for CRISPR-Cas system prediction using sequence encoding techniques (This figure was drawn using free version of BioRender; https://www.biorender.com).

To serve as input for AI models, sgRNA–DNA sequence data must first undergo preprocessing, commonly referred to as “sequence encoding”. This step transforms nucleotide sequences, composed of letters (A, C, G, T), into numerical formats that ML and DL algorithms can process and interpret (**Figure 5**). Proper encoding is critical for enhancing predictive accuracy. The two most widely adopted encoding methods in CRISPR-Cas9 research are (i) In one-hot encoding, each nucleotide is represented as a binary vector; e.g., A = [1, 0, 0, 0], C = [0, 1, 0, 0]), forming a 4×L matrix for a sequence of length L. Chuai et al. (2018) introduced DeepCRISPR, augmenting the standard one-hot encoding to a (4+n)×23 matrix by appending *n* epigenetic features per position, optimized for convolutional denoising networks. Lin and Wong (2018) proposed a unified 4×23 matrix encoding both sgRNA and target DNA for use in feedforward and convolutional networks. Charlier et al. (2021) developed a bijective mapping combining separate 4×23 matrices into an 8×23 input for FNNs, CNNs, and RNNs. Zhang et al. (2020a) further expanded the encoding to a 20×L multi-channel matrix incorporating bulges and mismatches, improving off-target prediction with CNNs and data augmentation. (ii) Word embedding, by contrast, assigns each substring (or *k*-mer) a dense vector in a continuous space, capturing semantic or contextual relationships between sequences. A widely used embedding method is Word2Vec (Mikolov et al., 2013), a neural network-based technique originally developed for natural language processing. Liu et al. (2020a) combined word embedding with transformers, CNNs, and FNNs, achieving performance comparable to advanced one-hot methods. They later used GloVe to generate dense sgRNA vectors fed into bidirectional LSTM and CNN models, reaching state-of-the-art off-target prediction.

**Figure 6:**
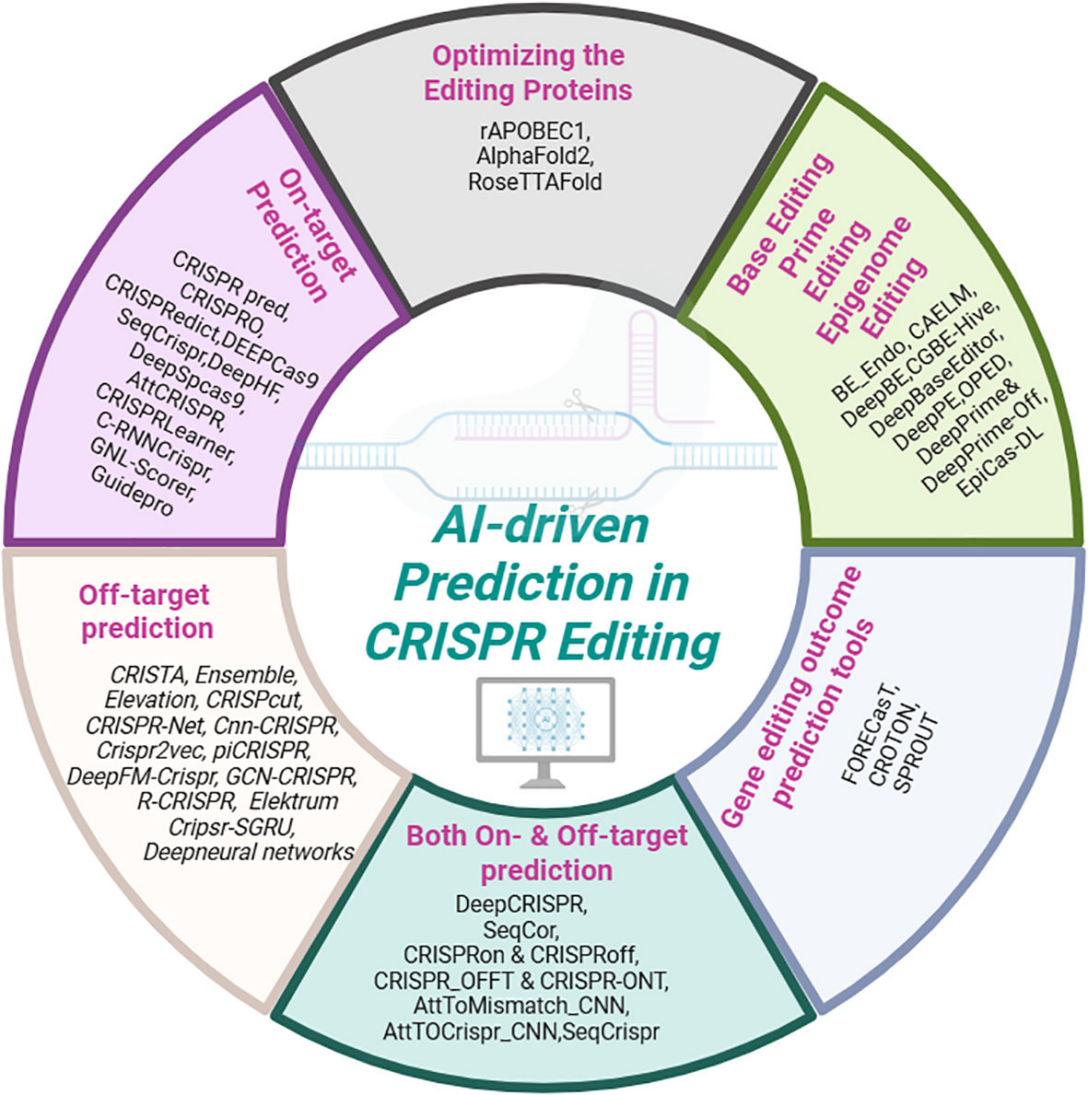
AI-driven prediction tools and applications in CRISPR genome editing (This figure was drawn using free version of BioRender; https://www.biorender.com). .

Beyond encoding, traditional ML models rely on engineered features such as nucleotide frequencies, GC content, thermodynamic properties (Doench et al., 2014; Xu et al., 2015), RNA secondary structure and n-gapped dinucleotides (Rahman and Rahman, 2017). Biological annotations like exon positions, amino acid traits, and domain occupancy also enhance predictions (Schoonenberg et al., 2018). Studies have highlighted the importance of positional and thermodynamic features, structural elements, accessibility, mismatch counts, and allele information (Muhammad Rafid et al., 2020). Statistical methods identify key predictors (Hiranniramol et al., 2020), and factors like frameshift probability and amino acid sensitivity improve sgRNA efficiency forecasts (He et al., 2021). Although deep learning models can automatically learn feature representations, manually engineered features remain essential for the performance of conventional machine learning models (**Figure 6**).

#### 4.2.2 Off-target prediction in CRISPR/Cas9 editing

In CRISPR gene editing, the single guide RNA (sgRNA) directs the Cas9 protein to a specific genomic site for targeted modification. However, Cas9 can sometimes cleave unintended sites, leading to off-target effects that may disrupt gene sequences and interfere with normal gene function. These unintended effects are influenced by factors such as the structure and length of the sgRNA (Chao and Fei, 2023). Off-target effects are typically categorized into mismatches between the sgRNA and DNA, RNA bulges (insertions), and DNA bulges (deletions). To mitigate off-target activity, AI-based prediction models are employed, primarily using two approaches. In the classification approach, genomic sites are labeled as “1” for off-target and “0” for on-target or non-off-target sites. In the regression approach, a continuous score is assigned to each site, representing the likelihood or severity of off-target activity. Reliable benchmark datasets are essential for training and evaluating these prediction models (**Table 3**).

**Table 3:** A summary of studies applying traditional machine learning and deep learning models/methods for off-target prediction in CRISPR/Cas9 editing.

ML/DL Types	Method	AI Model(s)	Encoding	Prediction metric values	Reference
Traditional machine learning	CRISTA	RF-based regression	GC content; sgRNA secondary structure	Spearman: 0.81; AUROC: 0.96; AUPRC: 0.96; R²: 0.8	Abadi et al., 2017
	Ensemble	SVM	One-hot; GC content; Position specific features	AUROC: 0.99; AUPRC: 0.45	Peng et al., 2018
	Elevation	Boosted regression, L1 regression, Naïve Bayes	One-hot	AUROC: 0.98	Listgarten et al., 2018
	Logistic Regression	SVM, RF, NN	One-hot; Mismatch position	Accuracy: 94%	Chen et al., 2019
	Ensemble	AdaBoost	One-hot; GC counts; Position specific features	AUROC: 0.938; AUPRC: 0.299	Zhang et al., 2019
	CHANGE-Seq	GTB	One-hot; Sequence features	AUROC: 0.995; AUPRC: 0.881	Lazzarotto et al., 2020
	CRISPcut	L1 logistic regression, L2 logistic regression, RF, XBoost	One-hot; GC counts; Position specific features	AUROC: 0.97; Accuracy: 91.49%	Dhanjal et al., 2020
Deep learning	Deep Neural Networks	CNN & FNN	One-hot	AUROC: 0.97 CNN, 0.97 FNN	Lin and Wong, 2018
	CRISPR-Net	LRCN	One-hot; sequence features	AUROC: 0.995	Lin et al., 2020c
	Cnn_Crispr	BLSTM CNN	Embedding GloVe	AUROC: 0.957; AUPRC: 0.429	Liu et al., 2020a
	DNA-BERT and Light GBM	Classification & Regression	Embedding	AUROC: 0.993; AUPRC: 0.594; Spearman: 0.276	Chen et al., 2019
	DL-CRISPR	Data augmentation	One-hot	Accuracy: 98.57%; Sensitivity: 95.13%	Zhang et al., 2020b
	Deep Neural Networks	FNN, CNN, RNN, RF, NB, LR	One-hot	AUROC: 0.995; AUPRC: 0.949	Charlier et al., 2021
	piCRISPR	RNN, CNN	One-hot; Physical features; target guide; target mismatch	AUROC: 0.983; AUPRC: 0.978; Spearman: 0.1	Störtz and Minary, 2021
	R-CRISPR	bi-directional recurrent network	One-hot	AUROC: 0.991; AUPRC: 0.319	Niu et al., 2021
	GCN-CRISPR	Graph Convolution Network	One-hot	AUROC: 0.987	Vinodkumar et al., 2021
	CRISPR-IP	CNN, BLSTM	One-hot	AUROC: 0.982; AUPRC: 0.751	Zhang and Jiang, 2022
	Elektrum	Transfer learning, KINNs	One-hot	AUPRC: 0.324	Zhang et al., 2023
	DeepFM-Crispr	Transformer-based DL + LLM	One-hot; Secondary structure	AUPRC: 0.69	Bao and Liu, 2024
	Crispr-SGRU	Stacked BiGRU + Inception	One-hot	AUROC: 0.999; AUPRC: 0.895	Zhang et al., 2024

RF, Random Forest; SVM, Support Vector Machine; NN, Neural Networks; GTB, Gradient Tree Boosting; CNN, Convolutional Neural Network; FNN, Feedforward Neural Network; LRCN, Long-term Recurrent Convolutional Network; BLSTM, Bidirectional Long Short-Term Memory; KNN, K-Nearest Neighbors; NB, Naïve Bayes; LR, Logistic Regression; LLM, Language Learning Model; AUROC, Area Under the Receiver Operating Characteristic Curve; AUPRC, Area Under the Precision-Recall Curve.

**Benchmark dataset and prediction algorithms**

GUIDE-seq, developed by Tsai et al. (2015), was one of the first genome-wide methods for off-target detection and remains a benchmark. Using sgRNAs targeting vascular endothelial growth factor (VEGFA), fanconi anemia -associated gene (FANCF), and HEK293 loci, it identified 28 off-target sites (≥0.1% modification) from 403 candidates. Later, Tsai et al. (2017) introduced CIRCLE-seq, a high-throughput *in vitro* method that profiled 10 gRNAs and detected 7,371 active off-targets, including mismatches, insertions, and deletions. CRISTA (Abadi et al., 2017) is a random forest-based method that uses features such as genomic rigidity and PAM proximity to predict Cas9 cleavage sites. The model was trained on datasets from three unbiased genome-wide profiling methods: GUIDE-seq (Tsai et al., 2015), HTGTS (Frock et al., 2015), and BLESS (Ran et al., 2015; Slaymaker et al., 2016), covering 33 sgRNAs and 872 confirmed off-targets. Peng et al. (2018) utilized datasets comprising 215 and 527 sgRNA–DNA pair, derived from data sources such as GUIDE-seq (Tsai et al., 2015), HTGTS (Frock et al., 2015), CIRCLE-seq (Tsai et al., 2017), and Digenome-seq (Kim et al., 2015, 2016). To handle class imbalance, they applied under-sampling and trained an ensemble SVM classifier, achieving superior off-target prediction performance. Listgarten et al. (2018) developed *Elevation*, a two-layer regression model for gRNA–target scoring. The first layer predicts off-target activity for single mismatches; the second uses penalized linear regression to combine multiple mismatches. Guide-level scores are generated by aggregating target scores via boosted regression trees, incorporating features like gene context to improve accuracy.

CRISPEY (Chen et al., 2019) dataset consisting of 23,936 samples, distinguishing 306 effect and 23,630 no-effect entries. They evaluated logistic regression, SVM, random forest, and DNN models, with SVM achieving the highest recall (64%) and logistic regression showing the highest accuracy (94%). Zhang et al. (2019) used an ensemble learning approach that combined scores from tools like CCTop (Stemmer et al., 2015), MIT (Haeussler et al., 2016), CFD (Doench et al., 2014), and Cropit (Singh et al., 2015), along with chromatin and conservation data. Their *AdaBoost* based model demonstrated superior performance on a dataset of 25,332 candidates, including 152 confirmed off-targets. Lazzarotto et al., 2020 trained a gradient boosting model using encoded vectors derived from their CHANGE-seq data. Their model emphasized PAM and protospacer features and outperformed GUIDE-seq and CIRCLE-seq in terms of specificity. Lin and Wong (2018) applied transfer learning to improve predictions on GUIDE-seq data using a model trained on CRISPOR data. While their initial encoding method (a 4×23 matrix) resulted in information loss, a subsequent improvement with a 7×23 lossless encoding significantly boosted model performance. In a follow-up study by Lin et al., 2020c, the authors introduced CRISPR-Net, an LRCN model with inception modules and bidirectional LSTM layers, which further improved off-target prediction accuracy.

Ji et al. (2021) developed DNA-BERT by adapting the original BERT model for DNA sequence analysis. To overcome limited training data, they pretrained DNA-BERT on extensive genomic datasets. The resulting embeddings were combined with handcrafted features, including mismatches and secondary structure, and used with LightGBM to build classification and regression models. DL-CRISPR (Zhang et al., 2020b), a deep learning framework which tackled data imbalance using a novel augmentation method and an ensemble strategy. Charlier et al. (2021) proposed a novel 8×23 matrix encoding for sgRNA–DNA pairs, testing various FNN, CNN, RNN, and traditional classifiers (e.g.,Random Forest, naive Bayes, logistic regression). Using transfer learning on GUIDE-seq and CRISPOR datasets, their framework improved prediction accuracy, raising AUCROC scores by up to 35% over previous methods. Störtz and Minary (2021) introduced piCRISPR, incorporating physically informed features through four encoding schemes (target-guide, target-mismatch, mismatch-type, and target-OR-guide, and used SHAP to evaluate feature importance. Their results on the crisprSQL dataset highlighted the roles of sequence context and chromatin accessibility in cleavage prediction. Niu et al. (2021) proposed R-CRISPR, encoding sgRNA targets into binary matrices fed into a CNN with Rep-VGG layers, followed by bidirectional LSTM for precise off-target prediction.

CRISPR-IP (Zhang and Jiang, 2022) is a deep learning model designed to enhance off-target activity prediction by encoding detailed sequence pair information. It employs a novel encoding scheme that separates functional regions through a function channel and distinguishes bases from base pairs using type channels. The model integrates CNN, BiLSTM, and attention mechanisms to effectively extract features from the encoded sequences. Elektrum (Zhang et al., 2023) is a deep learning framework for modeling biochemical reaction kinetics with high accuracy and interpretability. It first trains kinetically interpretable neural networks (KINNs) on *in vitro* assay data to predict reaction rates. These KINNs are then embedded into deeper convolutional networks and fine-tuned via transfer learning to predict *in vivo* outcomes. Crispr-SGRU (Zhang et al., 2024) is a deep learning model for predicting off-target activity with mismatches and indels. It combines inception and stacked BiGRU architectures and uses a dice loss function to handle class imbalance. Interpretability analyses via Deep-SHAP and knowledge distillation reveal that the model effectively captures sequence patterns linked to off-target effects. Bao and Liu (2024) developed DeepFM-Crispr, as a versatile and robust deep learning model, originally developed for the Cas13d system but adaptable to other CRISPR-Cas platforms. By leveraging large language model techniques, it captures complex genetic interactions and sequence features critical for accurate genome editing.

#### 4.2.3 On-target prediction in CRISPR/Cas9 editing

When the guide RNA (gRNA) is designed to target a specific DNA sequence, it forms a complex with the Cas9 protein, guiding the CRISPR system precisely to that genomic locus. Upon binding, Cas9 induces a double-stranded break (DSB) at the targeted site. This break initiates the cell’s intrinsic DNA repair mechanisms, predominantly through two pathways: non-homologous end joining (NHEJ), a quick but error-prone repair process that directly ligates the broken DNA ends, often resulting in small insertions or deletions (indels) at the cleavage. In contrast, homology-directed repair (HDR) uses a homologous DNA template to accurately repair the break, allowing precise editing but generally occurring at lower efficiency (Lackner et al., 2023). While these repair mechanisms enable genome editing, they also pose significant challenges due to their variability and unpredictability, which can lead to unintended genetic alterations at the target site. To mitigate these issues and enhance the precision of genome editing, AI approaches have been developed to predict the efficiency and specificity of gRNAs, thereby forecasting their on-target activity and minimizing off-target risks (**Table 4**).

**Table 4:** A summary of studies applying traditional machine learning and deep learning models/ methods for on-target prediction in CRISPR/Cas9 editing.

ML/DL Types	Method	AI Model(s)	Encoding	Prediction metric values	Reference
Traditional machine learning	ML	SVM	One-hot; GC-content	log2 fold change	Wang et al., 2014
	ML	SVM & Logistic regression	One-hot; GC-content; Position specific feature	AUROC: 0.8	Doench et al., 2014
	ML	Logistic regression	One-hot; GC-content; Position specific feature	AUROC: 0.73	Xu et al., 2015
	ML	SVM, L1 regression, L2 regression, RF regression, SVM + logistic regression, Linear regression, GBRT	One-hot; GC-content; Position specific feature	Spearman: 0.52; AUROC: 0.75	Listgarten et al., 2018
	CRISPR pred	SVM, RF, Liner regression	One-hot; Position specific-feature; Position-independent feature; Structural/thermodynamic feature	AUROC: 0.85; AUPRC: 0.56; MCC: 0.4	Rahman and Rahman, 2017
	CRISPRO	GBDT, Ridge, RF, Lasso, SVM	One-hot; GC-content; Position specific feature	Spearman: 0.57	Schoonenberg et al., 2018
	CRISPRpred (SEQ)	SVM	Position specific feature; Position independent feature; n-gapped di-nucleotide	AUROC: 0.893; Spearman: 0.829	Muhammad Rafid et al., 2020
	Guidepro	Two layer ensemble SVM and RF	Sequence specific feature	Spearman: 0.523	He et al., 2021
	GNL-Scorer	GBRT, DT, Linear regression, L2 regression, L1 regression, BRR, RF, NN	One-hot; GC-count; Position independent; Position dependent features; Thermodynamic features	Spearman: 0.502	Wang et al., 2020
	sgDesigner	Stacking SVM and XGBoost using logistic regression	GC-content; Structural features	Spearman: 0.75; AUROC: 0.934; Accuracy: 86.3%	Hiranniramol et al., 2020
	BoostMEC	Boosting	GC-content; Position-specific features; Thermodynamic features	Spearman: 0.78	Zarate et al., 2022
	CRISPRedict	Linear regression, Binomial regression, Logistic regression	Position-specific nucleotide composition; Structural properties of sRNAs	Spearman: 0.380 for U6 data sets, and 0.355 for T7 data sets	Konstantakos et al., 2022
Deep learning	DeepCas9	CNN	One-hot	Spearman: 0.23-0.61	Xue et al., 2019
	SeqCrispr	RNN + CNNs + transfer learning	Embedding	Spearman: 0.77	Liu et al., 2019
	DeepHF	RNN	Embedding; GC-content; Position-specific features; Position independent features; Thermodynamic feature	Spearman: 0.867	Wang et al., 2019
	DeepSgRNA	CNN	One-hot	Spearman: 0.82; AUROC: 0.85	Shrawgi and Sisodia, 2019
	CRISPRLearner	Deep CNN & Data augmentation	One-hot	Spearman: 0.23-0.69	Dimauro et al., 2019
	DeepSpCas9	CNN	One-hot	Spearman: 0.73	Kim et al., 2016
	C-RNNCrispr	CNN & RNN	One-hot	AUROC: 0.976; Spearman: 0.877	Zhang et al., 2020a
	AttCRISPR	CNN & RNN	One-hot; Embedding	Spearman: 0.872	Xiao et al., 2021
	DeepCRISTL	BLSTM + transfer learning	Embedding; GC-content; Position-specific features; Position independent features; Thermodynamic features	Spearman: 0.878	Elkayam et al., 2024
	DeepMEns	CNN, Transformer, LSTM, Attention-Mechanism	One-hot; Secondary structure features; Position encoding	Spearman: 0.403	Ding et al., 2025

RF, Random Forest; SVM, Support Vector Machine; ML, Machine learning; GBRT, Gradient Boosted Regression Trees; DT, Decision tree; BRR, Bayesian ridge regression; NN, Neural Networks; LSTM, Long Short-Term Memory; BLSTM, Bidirectional Long Short-Term Memory; CNN, Convolutional Neural Network; RNN, Recurrent Neural Network; MCC, Matthews Correlation Coefficient; AUROC, Area Under the Receiver Operating Characteristic Curve; AUPRC, Area Under the Precision-Recall Curve.

**Benchmark dataset and prediction algorithms**

Early machine learning approaches for on-target prediction include Wang et al. (2014), employed SVMs with fold-change data to classify sgRNA efficacy from a library of 73,000 sgRNAs targeting ribosomal and non-ribosomal genes in HL-60 and KBM7 cells; Doench et al. (2014), used logistic regression to distinguish top and bottom activity quintiles and Xu et al. (2015), applied elastic-net regression, outperforming previous models. The studies from Rahman and Rahman, 2017 and Muhammad Rafid et al., 2020, emphasized feature engineering’s importance, developing SVM-based tools (CRISPRpred and CRISPRpred(SEQ)) that rival deep learning models like DeepCRISPR. CRISPRO, a computational pipeline developed by Schoonenberg et al. (2018), links functional scores of guide RNAs to genomic, transcript, and protein-level coordinates and structures. Xue et al. (2019) introduced DeepCas9, a CNN-based framework outperforming traditional methods. DeepHF method was used to generate the largest dataset, measuring indel rates of over 50,000 gRNAs across ~20,000 genes for three SpCas9 variants (Wang et al., 2019). Deep learning advancements include the DeepSgRNA CNN model, which eliminates manual feature engineering and achieves state-of-the-art accuracy on GenomeCRISPR data (Shrawgi and Sisodia, 2019).

CRISPRLearner (Dimauro et al., 2019) utilizes a deep convolutional neural network to automatically extract sequence features and predict sgRNA efficiency. It can operate using either pre-trained models or be trained on custom datasets. The CNN employs linear regression to estimate efficiency from the training data, with ten different models developed across ten unique gene datasets. GuidePro, a two-layer ensemble model was introduced by He et al. (2021) to prioritize sgRNAs for protein knockout by integrating diverse predictive features. When tested on independent datasets, GuidePro consistently outperformed existing tools in predicting protein loss-of-function phenotypes, highlighting its reliability and effectiveness across various CRISPR/Cas9 knockout applications. GNL-Scorer is used for cross-species sgRNA activity prediction by combining multiple datasets and models (Wang et al., 2020). Hiranniramol et al. (2020) developed the sgDesigner plasmid library, consisting of 12,472 oligonucleotides, to assess sgRNA efficiency using machine learning. Zarate et al. (2022) developed BoostMEC, a LightGBM-based gradient boosting model that outperformed many deep learning methods while providing greater interpretability.

CRISPRedict an interpretable web tool employing regression models and nucleotide composition features, achieving competitive accuracy (Konstantakos et al., 2022). C-RNNCrispr, a hybrid model introduced by Zhang et al. (2020a), integrates convolutional neural networks (CNNs) and bidirectional gated recurrent units (BGRUs) to predict CRISPR/Cas9 sgRNA on-target activity. The model has two branches, an sgRNA branch and an epigenetic branch—that process a binary-encoded sgRNA sequence and four epigenetic features, respectively, ultimately producing a regression score. AttCRISPR, a deep learning approach designed for intrinsically interpretable on-target activity prediction. By combining encoding-based and embedding-based methods through an ensemble learning strategy, it improves both interpretability and prediction accuracy (Xiao et al., 2021). DeepMEns, is a deep learning ensemble model for predicting sgRNA on-target activity (Ding et al., 2025). It combines five sub-regression models, each with three components utilizing different encoding methods and neural network architectures. By integrating multi-feature representation, ensemble learning, and attention mechanisms, DeepMEns achieved superior performance on three independent test sets, WT-SpCas9, eSpCas9(1.1), and SpCas9-HF1.

#### 4.2.4 Both on- and off-target prediction in CRISPR/Cas9 editing

The benchmark datasets included in this context contains both on-target and off-target information (**Table 5**). Doench et al. (2016) developed optimized genome-wide sgRNA libraries for human and mouse using machine learning models to predict on- and off-target activity. Evaluated models included linear regression, L1/L2-regularized regression, L1 logistic regression, linear-kernel SVM, hybrid SVM-logistic regression, random forest, and gradient-boosted regression trees. DeepCRISPR dataset introduced by Chuai et al. (2018), has approximately 680 million sgRNA sequences from 13 human cell lines (e.g., HEK293, MCF-7, K562, HCT116), enriched with cell type–specific epigenetic information. They also developed DeepCRISPR, a deep learning framework capable of jointly predicting on-target knockout efficiency and off-target cleavage. The model utilizes a deep convolutional denoising neural network (DCDNN) for feature extraction, followed by a hybrid neural network that performs both classification (via softmax) and regression (via an identity function). Liu et al. (2019) introduced two transformer-based models, AttnToMismatch_CNN for off-target specificity and AttnToCrispr_CNN for on-target efficiency. Key innovations include attention-based architectures for sequential genomic data, integration of cell-specific network-based gene properties, a novel matrix encoding for sgRNA–DNA pairs, and a universal feature ranking algorithm for model interpretability. SeqCor, an open-source tool based on random forest, designed to extract sequence features affecting both sgRNA efficiency and off-target activity, while minimizing bias from library design (Liu et al., 2020b). Two attention-based deep learning models: CRISPR-ONT for predicting sgRNA efficiency and CRISPR-OFFT for off-target specificity was developed by Zhang et al. (2021). CRISPR-ONT combines a CNN with attention to emphasize PAM-proximal regions crucial for Cas9 cleavage. CRISPR-OFFT integrates CNN and attention to extract multi-level features from sgRNA-DNA pairs for off-target prediction. Xiang et al. (2021) developed CRISPRon and CRISPRoff, two deep learning models designed for predicting gRNA activity. CRISPRon estimates on-target efficiency for gRNAs with NGG PAM sequences, while CRISPRoff evaluates specificity and the relative likelihood of off-target cleavage. Both models combine convolutional neural networks (CNNs) with gradient boosting trees and incorporate position-specific, position-independent, and thermodynamic features.

**Table 5:** A summary of studies applying traditional machine learning and deep learning models/methods for on- and off-target prediction in CRISPR/Cas9 editing.

ML/DL methods	Method	AI Model(s)	Encoding	Prediction metric values	Reference
Machine learning/Deep learning methods for on‐ and off‐target prediction in CRISPR/Cas9	ML/ANN	Boosted RT, L1 regression, L2 regression, SVM + Logistic regression	One-hot; GC-counts; Position specific features; Position-independent features; Thermodynamic features	Spearman: 0.54 (on-target); AUROC: 0.8 (off-target)	Doench et al., 2016
	DeepCRISPR	DCDNN	One-hot	Spearman: 0.246; AUROC: 0.804; AUPRC: 0.303	Chuai et al., 2018
	AttnToMismatch_CNN	Transformer + CNN	Embedding	AUROC: 0.961; AUPRC: 0.071	Liu et al., 2019
	AttnToCrispr_CNN	Transformer + CNN	Embedding	Spearman: 0.77; Pearson: 0.781; MSE: 412 ± 27	
	seqCrispr	LSTM + CNN	One-hot	Spearman: 0.765; Pearson: 0.760; MSE: 442 ± 33	
	SeqCor	RF	A general-purpose hash function	Spearman: 0.4 for off-targets and 0.369 for on-targets	Liu et al., 2020b
	CRISPRon & CRISPRoff	GBRT	One-hot; GC content; Position specific features; Position independent features; Thermodynamic features	Spearman: 0.91	Xiang et al., 2021
	CRISPR-OFFT	CNN, attention	Embedding	AUROC: 0.97; AUPRC: 0.79	Zhang et al., 2021
	CRISPR-ONT	CNN	Embedding	AUROC: 0.865	

DCDNN, Deep Convolutional Denoising Neural Network; LSTM, Long Short-Term Memory; GBRT, Gradient Boosted Regression Trees; CNN, Convolutional Neural Network; RF, Random Forest; SVM, Support Vector Machine; AUROC, Area Under the Receiver Operating Characteristic Curve; AUPRC, Area Under the Precision-Recall Curve; MSE, Mean Squared Error.

#### 4.2.5 Performance metrics for on-target and off-target detection

Accurate evaluation of on-target and off-target effects in CRISPR/Cas9 systems relies on robust performance metrics. Commonly used measures include AUPRC (Area under precision recall curve), PR-AUC (Precision recall area under curve), MCC (Matthews correlation coefficient), Spearman and Pearson correlations, and the CFD (Cutting frequency determination) score, each with specific strengths and limitations. AUC-ROC (Area under the receiver operating characteristic curve metrics) provides an overall measure of classification performance but may be less informative in highly imbalanced datasets. PR-AUC, by contrast, is more effective in highlighting rare off-target events due to its focus on precision and recall. MCC considers all elements of the confusion matrix and is well-suited for imbalanced data, though it can be sensitive to small sample sizes. Spearman and Pearson correlations assess the agreement between predicted and observed outcomes, with Spearman capturing monotonic trends and Pearson evaluating linear relationships; both, however, can be affected by outliers and skewed distributions. The CFD score, specific to CRISPR, estimates the likelihood of off-target cleavage but may not fully account for sequence context in rare events. A combined use of these metrics offers a more comprehensive assessment of CRISPR editing accuracy, particularly for detecting low-frequency off-target effects (Doench et al., 2014).

#### 4.2.6 Gene editing outcome prediction tools

About five computational tools have been developed for predicting CRISPR-Cas9 editing outcomes, each introducing novel benchmark datasets. inDelphi (Shen et al., 2018) was the first, predicting 90 microhomology (MH) deletions, 59 non-MH deletions, and 4 one-base insertions using data from 1,095 target sites across human and mouse cell lines (HEK293, K562, HCT116, mESCs, U2OS). FORECasT, predicts gRNA-induced mutations using synthetic constructs, with over 31,000 samples and ~440 mutation types (Allen et al., 2018). SPROUT, provides detailed editing outcome statistics, including indel rates, lengths, edit diversity, inserted bases, and efficiency (Leenay et al., 2019). CROTON, employs CNNs and neural architecture search to predict 1 bp indels, deletion frequencies, and frameshifts from raw sequences, trained on FORECasT and evaluated on SPROUT’s endogenous T-cell data (Li et al., 2021a). Apindel (Liu et al., 2022a) integrates FORECasT and Lindel data to predict 557 mutation types, covering insertions from 1 bp to ≥29 bp across multiple classes.

#### 4.2.7 Tools for optimizing the editing proteins

In addition to designing highly specific guide RNAs, off-target effects in genome editing can be reduced by modifying or discovering novel editing proteins. New bioinformatics approaches have led to the identification of new proteins like LrCas9 (Zhong et al., 2023), Cas13X, and Cas13Y (Xu et al., 2021). Protein engineering methods have further optimized existing editors such as VQR-Cas9 (Kleinstiver et al., 2015), SpRY (Walton et al., 2020), xCas9 (Hu et al., 2018), SpCas9-NG (Nishimasu et al., 2018), eSpCas9 (Slaymaker et al., 2016), LZ3 Cas9 (Schmid-Burgk et al., 2020) offering improvements over SpCas9 in terms of size, PAM compatibility, specificity, and efficiency. Many of these enhanced systems have already been applied successfully in plant genome editing (Hua et al., 2019; Li et al., 2021b). Traditional protein discovery and optimization methods are time-consuming and costly, often requiring detailed structural knowledge. However, advances in machine learning, particularly structure prediction tools like AlphaFold (Senior et al., 2020), AlphaFold2 (Jumper et al., 2021), and RoseTTAFold (Baek et al., 2021), have significantly improved the prediction of protein structures and functions. For example, AlphaFold2 was used to compare wild-type Cas12a with its active variants, revealing structural changes that explain functional differences and guiding the design of more specific Cas variants (Ma et al., 2022). Huang et al. (2023) applied AlphaFold2 to predict the structures of proteins containing deaminase domains, grouped them by structural similarity, and experimentally validated members of the SCP1.201 clade.

#### 4.2.8 Role of AI in enhancing base editing, prime editing and epigenome editing

In recent years, the development of ML and DL models has significantly advanced the ability to predict the outcomes and efficiency of base editing in CRISPR-Cas9 systems (**Table 6**). BE-DICT, an attention-based DL model that takes protospacer sequences as input and outputs editing probabilities per nucleotide, achieving AUC scores of 0.86 for ABEmax, 0.94 for CBE4max, 0.66 for ABE8e, and 0.97 for TargetAID (Marquart et al., 2021). Pallaseni et al. (2022) introduced an ML model that used DNA sequence and positional information to predict editing outcomes for various base editors. The model achieved precision scores between 0.49 and 0.72, and highlighted the challenge of predicting editing efficiency and bystander mutations due to strong sequence dependence around the target base. CAELM, proposed by Li et al. (2022), uses chromatin accessibility and sequence features to predict CBE efficiency. Because the available dataset was relatively small (1,134 targets), they opted for the XGBoost algorithm, which often outperforms deep networks on limited data. Their model achieved a pearson correlation of 0.64. Chen et al. (2022) introduced a new class of C•G-to-G•C base editors (CGBEs), with editing profiles evaluated across 10,638 genomic sites, enabling over 90% precision and up to 70% efficiency in correcting 546 disease-related transversion SNPs. Park and Kim (2023) presented DeepABE and DeepCBE, DL-based web tools for ABE and CBE outcome prediction.

**Table 6:** List of other CRISPR-derived DNA editing methods and their AI-associated prediction models.

Other CRISPR-derived DNA editing methods	AI-associated prediction models
Base editing	BE_Endo (Yuan et al., 2024), CAELM (Li et al., 2022), DeepBE (Kim et al., 2024), CGBE-Hive (Koblan et al., 2021), DeepBaseEditor (Song et al., 2020)
Prime editing	DeepPE (Kim et al., 2021), OPED (Liu et al., 2023), DeepPrime & DeepPrime-Off (Yu et al., 2023)
Epigenome editing	EpiCas-DL (Yang et al., 2022)

Several ML and DL models have been developed to predict the outcomes of prime editing (PED). Koeppel et al. (2023) analyzed factors influencing PED insertion efficiency and developed an ML model that incorporates features such as nucleic acid structure, insert length, secondary structure, and the expression levels of TREX1 and TREX2, enzymes known to degrade the 3′ DNA flap essential for successful insertions. Their findings showed that insertion efficiency is strongly influenced by the insert sequence’s length, composition, and structural properties. In addition, Mathis et al. (2023) introduced PREDICT, a deep learning model based on a recurrent neural network (RNN), trained on over 90,000 PED experiments. PREDICT accurately forecasts editing outcomes and rates for small genomic alterations, achieving Spearman correlation values of 0.85 for intended edits and 0.78 for unintended ones.

CRISPR/Cas-based epigenome editing is a powerful method for regulating gene expression without altering the underlying DNA sequence. This is achieved by targeting specific genomic regions with CRISPR/Cas systems fused to epigenetic modifiers (Goell and Hilton, 2021). Studies by Rauschert et al. (2020) and Machnicka and Wilczynski (2020) demonstrate how AI techniques can aid in interpreting epigenetic mechanisms and reconstructing the epigenetic code. To support CRISPR-mediated epigenome editing (epi-GED) specifically, dedicated AI tools are beginning to emerge. One such tool is EpiCas-DL (Yang et al., 2022), a deep learning model designed to predict the activity of sgRNAs used in epigenetic editing. EpiCas-DL incorporates four key epigenetic features, gene expression, DNA methylation, chromatin accessibility, and the distance to the transcription start site, to enhance prediction accuracy.

#### 4.2.9 Current research gaps and prospective advances in AI-driven genome editing

Current CRISPR/Cas9 prediction models primarily focus on SpCas9, limiting their applicability across other CRISPR systems. Generalization to proteins like SaCas9 or Cpf1 remains underexplored. Advanced architectures such as LSTM, GRU, transformers, and embedding-based methods (e.g; DNA2vec k-mer representations) (Ng, 2017), offer potential for learning universal patterns applicable across systems and broader genomic contexts, including non-coding regions and whole-genome analysis. Performance is constrained by limited, imbalanced, and poorly labeled datasets. Addressing off-target effect (OFTE) prediction remains challenging. Integration of multi-omics data (genomic, epigenomic, transcriptomic, proteomic) can enhance predictive accuracy, especially by capturing features like DNA methylation and chromatin accessibility (Chuai et al., 2018; Sun et al., 2024). Deep learning models, particularly deep neural networks (DNNs), are composed of multiple layers with numerous neurons, making the design of efficient architectures and the tuning of hyperparameters both critical and challenging tasks. To optimize model performance in CRISPR/Cas9 applications, techniques such as evolutionary strategies, random search, exhaustive grid search, and Bayesian optimization (Goodfellow et al., 2016; Cho et al., 2020) should be systematically explored. Alongside architectural improvements, enhancing the explainability and interpretability of these models has become increasingly important (Goodfellow et al., 2016). Recent methods (Chou et al., 2021; Vilone and Longo, 2021) aim to address this by providing better insights into model behavior, which is particularly valuable for understanding on- and off-target effects in genome editing, a key factor for clinical translation. In terms of input representation, feature engineering has shown strong potential in improving prediction accuracy.

Emerging approaches include the use of hybrid deep learning models (e.g., CNN–RNN combinations), epigenetic information, and advanced DL architectures such as transformers. Transfer learning and pretraining on large-scale datasets like encyclopedia of DNA elements (ENCODE) or genotype-tissue expression (GTEx) (The ENCODE Project Consortium, 2012) could reduce reliance on extensive CRISPR-specific annotations. Engineered features like epigenetic markers, microhomology properties, and RNA fold scores have proven useful (Wang et al., 2019; Shrawgi and Sisodia, 2019), while CNNs and LRCNs offer automated feature extraction directly from sequence data, reducing biases associated with manual selection (Lin et al., 2020c; Shrawgi and Sisodia, 2019; Niu et al., 2021). Tools like SHapley Additive exPlanations (SHAP) (Lundberg and Lee, 2017), Tree-SHAP (Lundberg et al., 2018), and Deep-SHAP (Lundberg and Lee, 2017) further support model interpretability by quantifying each feature’s impact using Shapley value-based approaches. Model reliability can also be improved through uncertainty quantification. This involves assessing aleatoric uncertainty (stemming from data noise) and epistemic uncertainty (from model limitations) (Mazoure et al., 2022).

## 5 Key notes for applying AI and machine learning in plant biotechnology

Effective deployment of AI and ML in plant biotechnology hinges upon rigorous data management and understanding the intrinsic characteristics of the datasets involved.

### 5.1 Data considerations for AI/ML in plant biotechnology

The quality and quantity of data critically determine the reliability and applicability of machine learning models in biological research. Large, diverse datasets are essential for training complex models, particularly deep learning architectures, to prevent overfitting and enhance generalization (Kaushik et al., 2025). Standardized data collection, aided by automated sensors and uniform protocols, ensures consistency and reduces variability from experimental or operator differences (Ibrahim et al., 2023). Proper sampling strategies, such as stratified sampling, capture sufficient diversity across genotypes and environments, minimizing bias and supporting robust model training. Accurate labeling is vital for supervised learning, and expert annotation with iterative validation reduces error propagation (Hesami and Jones, 2021).

Data preprocessing steps such as cleaning or noisy observations and handling outliers ensure dataset integrity, while normalization and feature scaling prevent dominance of large-magnitude features and accelerate model convergence. Partitioning data into training, validation, and test sets through cross-validation enables fair evaluation and tuning (Hesami et al., 2020a; Niazian and Niedbała, 2020). Effective feature engineering, including biologically meaningful variable extraction and appropriate categorical encoding (e.g., one-hot encoding of nucleotide sequences), enhances model interpretability and performance. Finally, interpreting model outputs using comprehensive metrics (Liu et al., 2020a). Addressing data imbalances and avoiding overfitting or confounding biases require continuous vigilance throughout model development and deployment.

### 5.2 Choosing the most appropriate AI/ML approach

Selecting an optimal AI/ML approach requires assessing data volume, type, complexity, and the desired outcome (Hesami et al., 2020a). In tissue culture optimization, such as media formulation or growth prediction, regression and ensemble methods like random forests offer robustness to nonlinear and multivariate relationships (Aasim et al., 2022). Simpler models (e.g., linear regression) suit smaller, well-curated datasets, providing interpretability and lower computational cost (Zarbakhsh et al., 2024). Conversely, complex or high-dimensional problems benefit from neural networks or hybrid models integrating evolutionary algorithms to address biological complexity and data heterogeneity. For classification and detection tasks, particularly those involving images, convolutional neural networks (CNNs) excel by capturing hierarchical spatial features (Shavindi et al., 2024). When data are limited, tree-based or probabilistic classifiers (e.g., decision trees, naïve Bayes) serve as interpretable, data-efficient alternatives (Aasim et al., 2023). Reinforcement learning holds promise for dynamic or sequential decision-making, though its application in plant tissue culture remains in early exploration (Nasti et al., 2024). Across all scenarios, model selection should account for computational scalability, interpretability, and the practicality of experimental validation. Hybrid workflows that couple AI models with domain expertise and iterative experimentation typically produce more reliable and impactful results.

Applying machine learning (ML) to CRISPR genome editing begins with clearly defining the problem type. Predicting on-target efficiency constitutes a regression task (Wang et al., 2014), while off-target prediction involves classification or ranking (Chao and Fei, 2023). sgRNA design is addressed through generative modeling or optimization, base-editing outcome prediction through multiclass classification, and Cas variant selection through classification or recommendation models (Hiranniramol et al., 2020). The choice of ML method depends on data size and nature. Sequence-based tasks typically use CNNs or transformers, feature-based analyses favor gradient boosting or random forests, structure-based studies employ graph neural Networks (GNNs), and optimization problems leverage reinforcement learning or genetic algorithms. Data representation is equally critical (Liu et al., 2020a). sgRNA sequences are encoded as one-hot vectors, k-mers, or learned embeddings for deep models; structural information is modeled as graphs for GNNs; and epigenetic or chromatin features are expressed as numerical vectors for tree-based or neural models (Chuai et al., 2018). PAM sequences and experimental outcomes, such as indel frequencies, are incorporated into regression or classification models as relevant predictors (Zhang et al., 2020a).

## 6 Applications of artificial intelligence in bioinformatics

Artificial intelligence (AI) has become an essential tool in bioinformatics and computational molecular biology. With a broad array of AI algorithms now available, researchers are able to develop systems to classify, analyze, and mine biological data, resulting breakthroughs in genomics, drug discovery, and personalized medicine. Deep learning models such as AlphaFold have revolutionized protein structure prediction (Palvadi and Kadiravan, 2025). AI also assists in analyzing genomic, transcriptomic, and metabolomic data to identify disease biomarkers and predict outcomes (Venkadesh et al., 2025). However, challenges such as data quality and interpretability remain, highlighting the need for continued development (Bukhari et al., 2025). Furthermore, the wide variety of available methods makes it difficult to select the most appropriate approach for a given dataset. Consequently, there is an increasing demand for tools that present data intuitively, providing context, accuracy estimates, and clear explanations. The central motivation behind these AI-driven approaches is to gain a deeper understanding of organismal evolution while managing the complexities of working with noisy or incomplete data.

## 7 Challenges and future directions

•While the integration of AI into plant tissue culture and genome editing holds immense potential, several challenges must be addressed. In plant tissue culture, major hurdles include genotype dependence, contamination risks, somaclonal variation, and the high cost and labor-intensive nature of maintaining sterile environments. These limitations hinder the consistent and scalable propagation of plant species. AI, particularly through machine learning models, has shown promise in optimizing media compositions, predicting shoot and root regeneration, and reducing empirical trial-and-error. Future directions in this area include the development of generalized predictive models that can adapt across species, the integration of multi-omics and sensor data for dynamic culture optimization, and the deployment of robotic systems for high-throughput automation (Williamson et al., 2021). However, the success of AI in tissue culture relies heavily on the availability of diverse, high-quality data and the development of user-friendly interfaces accessible to non-experts (Zhang et al., 2025).

•The AI driven methodologies would provide an opportunity to build end-to-end, data-driven pipelines that reduce the time and cost associated with plant transformation and trait development. However, challenges such as data heterogeneity, lack of standardization, high implementation costs, and regulatory uncertainties must be addressed. Developing AI models that can generalize across diverse genotypes, supporting open data sharing, and building interdisciplinary teams of plant scientists, engineers, and data experts are essential steps forward (Farrell et al., 2025; Finzel, 2025). Additionally, ethical concerns related to gene editing, especially in food crops, require transparent regulatory frameworks and public engagement (Harfouche et al., 2021).

•In CRISPR-based genome editing, AI contributes to guide RNA (gRNA) design, prediction of off-target effects, and understanding editing outcomes, all of which are crucial for enhancing editing precision and minimizing unintended changes (Han et al., 2025). Despite this, the effectiveness of CRISPR in plants is often limited by inefficient delivery systems, tissue culture-dependent regeneration processes, and the complexity of polygenic traits (Sebiani-Calvo et al., 2024; Rafiq et al., 2025). AI can accelerate trait discovery by analyzing multi-omics and phenotypic data to identify target genes and regulatory networks. Moreover, future innovations aim to reduce or eliminate the reliance on tissue culture altogether, enabling direct in planta editing. This would not only streamline workflows but also expand editing capabilities to recalcitrant species. Integration of AI across the editing pipeline, from gRNA design to phenotypic evaluation, can significantly enhance efficiency, especially when coupled with high-throughput imaging and omics-based phenotyping platforms (Kim et al., 2025).

•Looking ahead, future research directions include the development of explainable AI models tailored for tissue culture, transfer-learning or few-shot learning approaches to allow models to generalize between plant species, synthetic data and augmentation to overcome limited datasets. Tighter integration of AI with sensor/automation systems and Internet of Things (IoT) environments for real-time feedback in culture systems. The field is also moving toward cloud-based platforms for data/model sharing, which shall facilitate collaborative development and validation of AI-driven tissue culture workflows (Holzinger et al., 2023).

•There is limited research translating AI-driven innovations from controlled laboratory settings to real-world field applications, particularly under variable environmental conditions. Moreover, interdisciplinary collaboration between AI, plant physiology, and molecular biology is still limited, constraining system-level advancements. Finally, ethical and regulatory considerations surrounding AI in plant biotechnology such as societal impacts, biosafety, and governance, remain underexplored, highlighting the need for a more comprehensive and responsible approach to AI-driven plant sciences.

## 8 Conclusion

Most studies have focused on optimizing experimental conditions and improving automation and efficiency through AI-based models that refine media composition, growth parameters, and editing targets. AI can significantly enhance plant tissue culture methods by optimizing growth conditions, making the process more efficient and scalable. Given the complex and non-deterministic nature of tissue culture, ML and optimization algorithms have been effectively applied to fine-tune key parameters (Hesami and Jones, 2020b). Future research may focus on ML-based virtual simulations to reduce experimental time and accelerate crop improvement (Ray, 2019). However, challenges such as data availability, scalability, and regulatory frameworks must be addressed to fully realize AI’s potential in plant biotechnology. Automated AI-powered systems have further to be streamlined for monitoring and quality control, reducing human error and conserving resources. Gene editing has become increasingly accessible, and advancements in computing power and ML continue to simplify the process (Ran et al., 2013). ML/DL/ANNs have helped overcome key challenges in the CRISPR/Cas system by improving the accuracy of outcome predictions and reducing the need for extensive experimental optimization (Chuai et al., 2017). Enhanced protein structure and functional residue predictions have also lowered the resources required to develop more effective gene-editing tools (Huang et al., 2023).
